# The emergence of nitrification during DOM processing by marine microbial assemblages

**DOI:** 10.1371/journal.pone.0336919

**Published:** 2025-12-03

**Authors:** Kevin J. Flynn, Darren R. Clark, Karen Tait, Susan A. Kimmance, Elaine S. Fileman, Luca Polimene

**Affiliations:** 1 Plymouth Marine Laboratory, Prospect Place, Plymouth, United Kingdom; 2 Somerset Scientific Services, Unit 2a, Westpark, 26 Chelston, Wellington, United Kingdom; 3 College of Life and Environmental Sciences, University of Exeter, Exeter, United Kingdom; 4 European Commission, Joint Research Centre (JRC), Ispra, Italy; Wroclaw University of Environmental and Life Sciences, POLAND

## Abstract

Dissolved organic matter (DOM) production, its degradation, and nitrification, are crucial interconnected processes in the ocean. Nitrate produced by nitrification supports oceanic primary production. Primary production is the main source of DOM which, when remineralised by heterotrophic bacteria, liberates ammonium, the substrate supporting activities of nitrifying bacteria and archaea. However, the mechanisms underpinning the interplay between these processes, their succession and feedback, remain poorly understood. To elucidate these dynamics, we conducted two distinct DOM degradation experiments where natural marine microbial communities were exposed to microalgal-derived DOM. After 150 d of DOM degradation, we observed a strong consumption of ammonium and an increase in nitrate concomitantly with increased presence of nitrifying prokaryotes. The time course of this event suggests that, when ammonium is not limiting, nitrification in the oceans is regulated by competition between nitrifiers and heterotrophic bacteria with growth of the latter being more efficient when sufficient labile DOM is available. However, carbon limitation due to progressive consumption of labile DOM and enrichment of recalcitrant compounds transfers competitive advantage to nitrifiers, in this way contributing to the emergence of nitrification. This hypothesis is supported by numerical simulations showing an increasing dominance of nitrifying groups concomitantly with increasing DOM recalcitrance.

## Introduction

In the marine environment, most of the dissolved organic matter (DOM) pool is freshly produced by microalgae (phytoplankton, mixoplankton) through photosynthesis, during grazing by mixoplankton and zooplankton, and following viral-induced lysis [1]. Heterotrophic bacteria communities quickly use labile fractions of such DOM [2] leaving behind residual fractions that results in the DOM pool becoming increasingly recalcitrant (i.e., resistant to fast degradation) [3,4]. Recalcitrant DOM (rDOM), comprising 10,000’s of compounds typically weighing less than 1000 Daltons, may persist in the ocean for extended time scales (up to millennia) [1]. This process, leading to the sequestration of CO_2_ as rDOM, is termed the Microbial Carbon Pump [2]. During DOM degradation, nitrogen associated with the DOM is regenerated as ammonium. Ammonium-N is mostly reconverted to organic forms in the surface ocean where it is preferentially used as the nitrogen-source by microalgae. However, ammonium-N also supports nitrification, a process occurring especially under light limiting conditions, e.g., below the euphotic zone. Nitrification is itself an autotrophic growth mode, in which ammonium (actually, the unionized form, ammonia) and nitrite are oxidized to nitrite or nitrate, respectively, providing energy to support chemolithotrophic CO_2_ fixation [5].

While the microorganisms involved in nitrification are relatively well known as bacteria (ammonia oxidizing bacteria, AOB, and nitrite oxidizing bacteria NOB), archaea [5,6] (the most ubiquitous nitrifiers in marine waters [7]) and comammox bacteria [8], the environmental factors favoring nitrification in the oceans remain unclear [9–11]. We know, however, that the use of nitrification to fix CO_2_ is a metabolically expensive process [12,13] and, therefore, we can expect that nitrifiers are able to thrive only when the growth of prokaryotes displaying more efficient metabolic strategies that allow them to be better competitors for essential resources (e.g., macro and micronutrients) is limited. For example, growth of heterotrophic bacteria can be limited by the availability of labile carbon as observed in stream sediments where the addition of labile organic matter favors their growth at the expense of nitrifiers [14].

Here we test the hypothesis that the appearance of nitrification in ocean water is enabled by the progressive increase of the proportion of recalcitrant forms present in the DOM pool and the consequential decrease in the activity of heterotrophic bacteria which otherwise outcompete nitrifiers. We thus investigate the time course of DOM consumption and the emergence of nitrification, relating the latter to the declining availability of labile DOM rather than just the regeneration of ammonium [15,16]. The stimulation for this exercise is the observation that ammonium regeneration alone is not sufficient to explain the observed variability of nitrification rates in the ocean [9]. Yool et al. [9], analyzing large globally distributed datasets, observed a high variability (up to 4 orders of magnitude) of the rates of NH_4_^+^-specific nitrification. To test our hypothesis, here we report two experiments where we exposed natural communities of microbial plankton collected from shelf water to different concentrations of nutrients and microalgal-derived DOM. Bulk DOM and nutrients evolution were followed for up to 360 days. The experiments were conducted in darkness to isolate the dynamics of DOM degradation and nitrification from the potential continuing photosynthetic production of fresh DOM and thence the consumption of bacterial regenerated NH_4_^+^ by phototrophs. Our work thus replicates conditions where light-driven DOM production has stopped, as when surface waters containing DOM and near-surface microbial communities are advected/mixed into the sub-photic zone. To aid the interpretation of the results, we developed a numerical model describing changes in microbial community and DOM transformation to reproduce and further explore *in silico* the dynamics observed in the experiments.

## Materials & methods

### Overview of the experiments

To test our working hypothesis, we performed two experiments in which two natural microbial communities were exposed to a wide range of environmental conditions including different sources/concentration of phytoplankton-derived DOM, different temperatures and initial NH_4_^+^ concentrations. The DOM used in both experiments was intended to reflect a mixture of metabolites leaked from healthy cells, released by viral lysis, and/or originated from part disrupted/digested remnants following from zooplankton grazing. As such, this DOM is expected to be similar in structure and composition to the natural DOM which prokaryotes are exposed to in the ocean [17].

All experimental set up and sampling steps were conducted under aseptic conditions, in a bacteria-free laminar flow hood.

#### Experiment I.

For this experiment (Ex.I), we used DOM derived from axenic cultures of the phytoplankton diatom *Chaetoceros calcitrans* (strain CCMP1315), grown at 20°C in enriched seawater (120 µM nitrate, 15 µM phosphate, 120 µM silicate), following previously developed methods [18]. A total of 80L culture, at a final cell density of ca. 0.2 × 10^6^ cells mL^-1^, confirmed as being bacteria-free by analytical flow cytometry of the media and also of inoculated sterile DOM vessels to check for bacterial growth, was subjected to 3 cycles of freeze-thaw (−20°C). The resultant suspension was then filtered through Whatman GF/F and then GF-75 filters giving a DOM solution of ≈ 400µM DOM-C, which was then divided into 9 x 8.75L sterile portions each in 10L bottles.

Triplicate bottles were either run as controls (no bacterial additions), inoculated with *Alteromonas* (see [18] for strain details), or with a natural microbial community to give an initial cell abundance of ca. 0.3 × 10^6^ mL^-1^. The *Alteromonas* bottles were intended to act as intermediate controls against the community bottles, as these bacteria are only capable of restricted utilization of DOM and incapable of nitrification. Microbes for the natural community incubations were collected from the Western Chanel Observatory station E1 (water column depth: 72 meters, https://www.westernchannelobservatory.org.uk/) at 10 m depth. This seawater was filtered to remove larger particles (>3µm, Whatman GF8) allowing both prokaryotes and smaller protists (including grazers) through. This experiment was conducted at 20°C, following from our original work [18].

#### Experiment II.

Based on the design and the outcomes of Exp.I, we designed a second experiment to further explore the interactions of temperature, nutrients and DOM source on microbial population, DOM degradation and nitrification dynamics. Additionally, RNA analyses were added to this experiment to derive insights on the temporal evolution of the prokaryote community. An overview of the controls and treatments for these incubations is shown in Table 1, with more detail in Table S1 (S1 Appendix).

**Table 1:** Overview of Exp.II. ‘●’ indicates additions; no DOM additions were made to ‘Controls’, so these cultures relied only on the DOM already present in the seawater. Treatments (‘Treat’) included additional DOM derived from freeze-thawed *Emiliania huxleyi* cells. Inorganic additions were, respectively, for NO_3_^-^, NH_4_^+^, PO_4_^3-^: ‘●’ 20, 15, 2.2 µM; ‘●●’ 20, 75, 5.9 µM. See also Table S1 (S1 Appendix).

Sub-Experiment	Control				DOM Treatment			
	Name	Natural microbial community + seawater	Extra inorganic nutrient	Extra DOM	Name	Natural microbial community + seawater	Extra inorganic nutrient	Extra DOM
1	Con #1	●			Treat #1	●		●
2	Con #2	●	●		Treat #2	●	●	●
3	Con #3	●	●●		Treat #3	●	●●	●

For Exp.II, DOM was prepared from axenic cultures of the mixoplankton *Emiliania huxleyi* (strain CCMP379) grown at 14°C on seawater-based media with 50 µM NO_3_^¬^ and 4 µM PO_4_^3-^. Nine 10L volumes of culture were prepared, with a separate 2L vessel set-up and run in parallel to monitor cell counts so that the 10L vessels used to supply the biomass for the experimental DOM remained undisturbed. On day 8 (with ca. 0.7 × 10^6^ *E. huxleyi* cells mL^-1^), at which time the nutrients were just being exhausted, the 10L vessels were subjected to repeated freeze/thaw cycles (−20°C). The DOM enriched media (bringing the total to ca. 400µM DOC-C, which was double the DOM background in the source seawater) was prepared by sequential filtration (Whatman GF-D, GF-F, GF-75) and then pumped into the experimental treatment vessels (hereafter, ‘Treat’ bottles – Table 1). A separate series of 9x10L vessels were prepared as controls; these contained complete sterile seawater-based media, thus containing background DOM, but were not inoculated with DOM from lysed *E. huxleyi*. Selected bottles were amended, following measurement of the ambient nutrient concentrations, so that they contained either background inorganic nutrient levels (ca. 0.15, 2.5, 0.75 µM NO_3_^-^, NH_4_^+^, PO_4_^3-^ respectively), or enrichments of 20, 15, 2.2, or 20, 75, 5.9 µM NO_3_^-^, NH_4_^+^, PO_4_^3-^ respectively. Nitrite concentrations were <0.1 µM where no additional nutrients were added, and ca. 2 µM with added nutrients (Table S1 in S1 Appendix). All bottles were then inoculated with a natural community of microbes, prepared in the same way as for Exp.I but from a different sampling date.

### Sampling, DOM and nutrient analyses

The bottles were incubated (at either 20 or 10°C for Exp.I or Exp.II respectively) in darkness, with periodic aseptic removal of volumes of water for analysis. Sampling of Exp. I was interrupted by Covid-19 pandemic lock-down between days 11–46, but continued for 413 days. Exp. II continued for 360 days. Analyses included: inorganic nutrients (NO_3_^-^, NO_2_^-^, NH_4_^+^, PO_4_^3-^, by standard oceanographic segmented-flow methodologies); DOC and DON (contracted to Dennis Hansel, University of Miami, USA) [19] and microbe abundance (photoautotrophs and bacteria) using a FACScan flow cytometer (Becton Dickinson) equipped with a 15mW laser exciting at 488 nm and with a standard filter set up; protist zooplankton using a FlowCAM (Vs-IV) fitted with a 20x objective and 50µm flowcell [20].

### Microbial community structure and RNA analyses (Exp. II)

Water samples (200 mL) for 16S rRNA gene metabarcode sequencing were filtered onto 0.22 µm PES filters (Merck-Millipore, Gillingham, UK) using sterile equipment and stored at −80°C. Genomic DNA was extracted using the DNeasy PowerWater kit according to the manufacturer’s instructions (Qiagen, Manchester, UK). Concentration and viability were tested using Qubit (Thermo Fisher Scientific, Waltham, Massachusetts, USA) prior to sequencing. High-throughput DNA sequencing was performed by NU-OMICS (Northumbria University, UK) using the 16S rRNA gene V4/V5 PCR primer pairs 515F-Y (GTGYCAGCMGCCGCGGTAA) [21] and 806R (GGACTACHVGGGTWTCTAAT) [22]. Sequencing was performed on a MiSeq Personal Sequencer (Illumina, San Diego, CA, USA) using the V2 500 reagent kit. Demultiplexed paired end FASTQ files were analysed using QIIME2 [23], amplicon sequence variants (ASVs) generated using DADA2 [24] and taxonomy was classified using the Greengenes version 13_8 reference database [25]. Taxonomic abundance data are expressed as percentage abundance (%) enumerated from fractional abundances in sample libraries. All 16S rRNA gene sequence data can be viewed and downloaded from the NCBI Sequence Read Archive using the BioProject ID PRJNA1115048.

The effect of +DOM (‘Treat’) and NH_4_^+^ addition upon temporal changes in community composition was tested using ANOSIM in Primer-E [26]. Co-variance amongst major Classes and Orders was investigated using Type 3 Similarity Profile (SIMPROF) with clustering tests [27].

### Model simulations

A numerical model was constructed using the system dynamics, systems biology, approach supported by DRAMA [28]. Each prokaryote type was described in terms of four state variables for metabolic-C and core-C (which together describes whole organism C-biomass), N-biomass and P-biomass. Ratios between these state variables were used to modulate responses to nutrient stresses through (de)repression of resource acquisition mechanisms. The model was configured to represent a community of 3 functional types of prokaryotes: generalist r-select organisms only capable of exploiting highly labile DOM (akin to *Alteromonas* [18]), specialist K-select organisms capable of also exploiting complex poorly-labile DOM, and nitrifiers capable of collectively performing nitrification. The DOM description included highly labile and a dynamic representation of complex DOM (requiring extracellular digestion to liberate low molecular weight forms for transport) in which lability decreases with declining DOM N:C. Thus, DOM state variables for dissolved organic C, N and P were used to describe labile DOC (lDOC, C only), DOC requiring extracellular digestion (xdDOC, C only), free amino and nucleic acid (NAA, C and N), DOM requiring extracellular digestion (xdDOM, C and N), together each with P associated with NAA and xdDOM. Further details are given in S1 Appendix. The model was configured to reproduce the general abiotic experimental starting conditions and initial bacterial biomass levels to attain outputs consistent with empirical results. The main purpose of this modelling exercise was to complement the empirical results with simulated variables which were not measured such as DOM fractions based on recalcitrance, in this way helping to test our hypothesis.

## Results

### Exp. I

**Fig 1:**
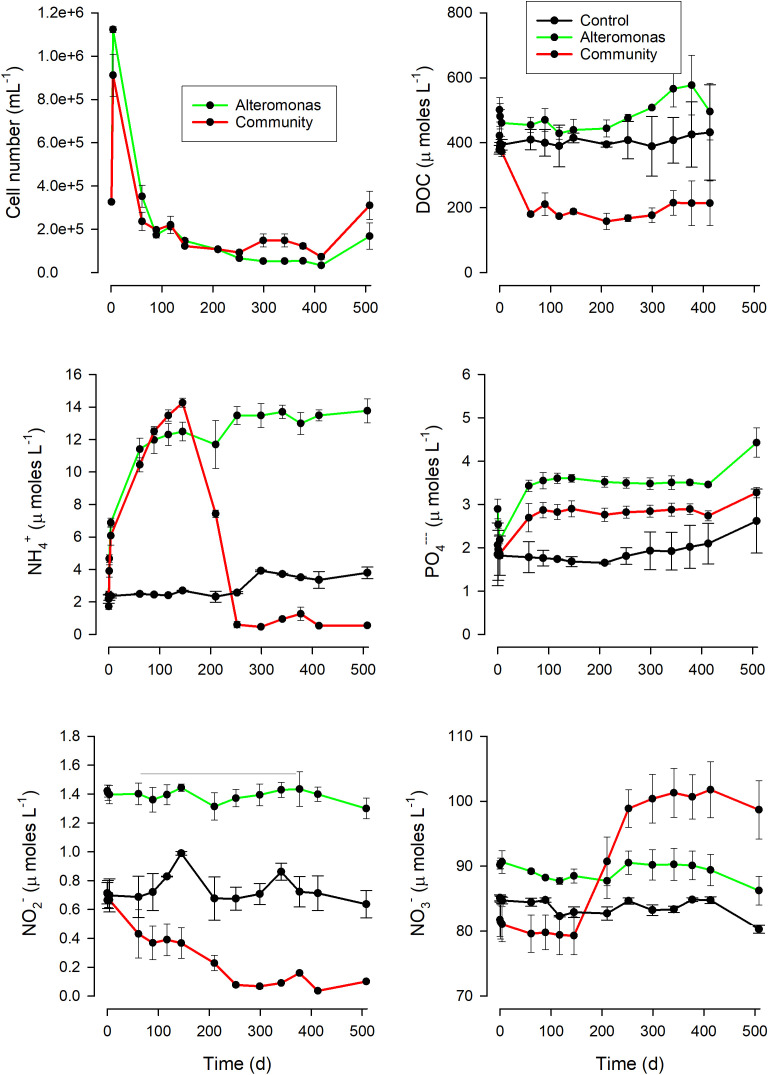
Microbial abundance, inorganic and organic nutrient concentrations in Exp.I. These values are averages across 3 bottles for each of the conditions, shown with confidence limits. Nutrients are shown for bottles labelled as ‘*control*’, ‘*Alteromonas*’ and ‘*community*’. See Fig S1 (S1 Appendix) for changes in the protist population structure in the ‘*community*’ bottles and Fig S2 (S1 Appendix) for changes in DOC content. Covid-19 restrictions prevented frequent sampling over the first month of the experiment.

Control bottles remained bacteria-free for all of the study, except for one bottle showing signs of contamination from day 200. As a results, both DOM and nutrients remained fairly constant for the duration of the experiment. The treatment bottles all showed a marked increase in abundance of bacteria/archaea within the first 4d and then later a decrease to below inoculation levels (Fig 1). Abundance of protists (assumed to be grazers of the prokaryotes) in the community bottles also showed a similar dynamic (Fig S1 in S1 Appendix).

The inorganic nutrient concentrations remained broadly constant in the control bottles, but there were contrasting changes in the ‘*Alteromonas*’ and ‘*community*’ bottles. The ‘*Alteromonas*’ bottles showed increases in NH_4_^+^ and PO_4_^3-^ but no changes in nitrite and nitrate. In contrast, while the initial trajectory of the ‘*community*’ bottles was the same as that of the ‘*Alteromonas*’ bottles, at around 150 d, a change occurred that saw a downturn in the concentration of NH_4_^+^ and an increase in NO_3_^-^ indicating a strong nitrification activity. It is noteworthy that there was a decline in NO_2_^-^ from the start of the experiment in the ‘*community*’ bottles, indicating the presence of a low level of nitrification from earlier in the incubations. The increase in NO_3_^-^ in excess of the net decrease in NH_4_^+^ in the ‘*community’* bottles (Fig 1) suggests a continual conversion of DOM-N to NH_4_^+^ concurrent with nitrification. The degradation of DOM in the treatment bottles appears to have been largely complete by this time, suggesting that the remaining DOM was functionally recalcitrant for the microbial community we have tested. The control and ‘*Alteromonas*’ bottles did not show significant evidence of net DOM degradation during the whole experiment, (Fig 1).

### Exp. II

The combinations of Exp.II treatments are given in Table 1, with target and actual initial nutrient concentrations provided in Table S1 (S1 Appendix).

**Fig 2:**
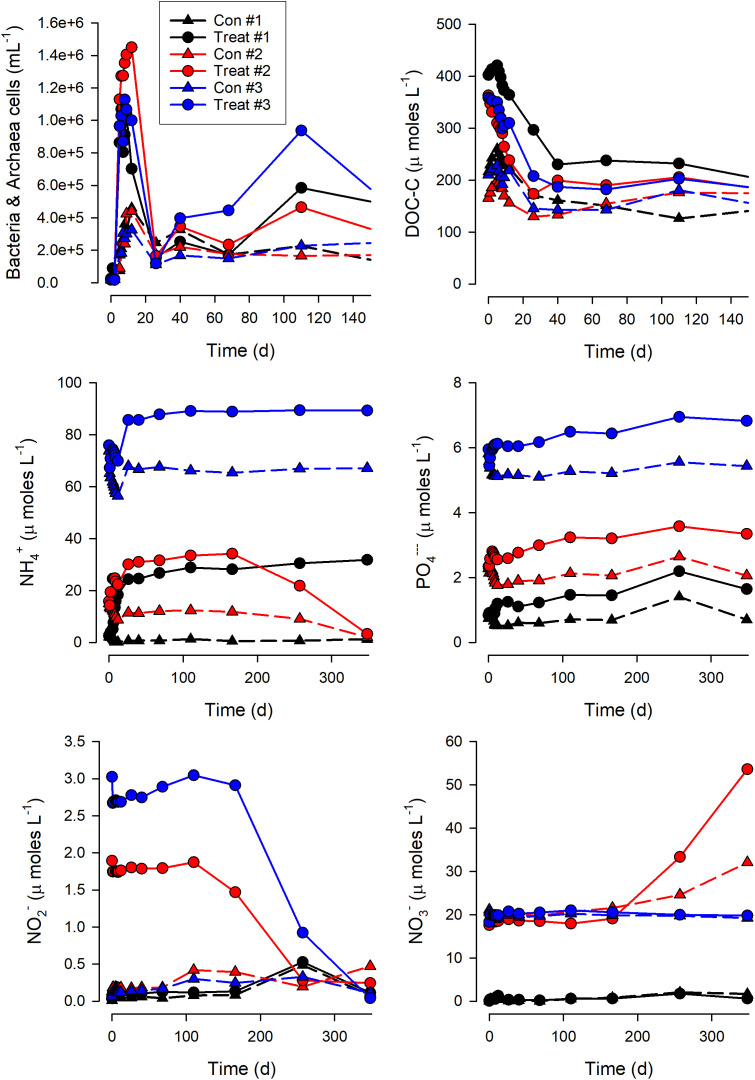
Microbial abundance, inorganic and organic nutrient concentrations in Exp.II. Values for each of 3 bottles for each condition (Table S1 in S1 Appendix). See Figs S4, S5 (S1 Appendix) for changes in the protist population structure, and Fig S6 (S1 Appendix) for addition plots for nutrients and for pH. For clarity, error bars from the triplicates are not shown around the sample data points; the relative magnitude of these errors were similar to those shown in Fig 1 for the respective data types.

The addition of fresh DOM prompted the growth of elevated microbial abundances (Fig 2) far in excess of that expected given the doubling in the bulk DOC concentration from that present in the seawater used as the base for the experiment media (Fig 2). The control cultures also increased in microbe abundance (bacteria/archaea, and their allied protist grazers – Fig S4 in S1 Appendix) and decreased the baseline DOM; this was so even in the treatment with no nutrient additions (Treat #1) but was greater with nutrient additions. Nutrient additions also enhanced microbial growth in the + DOM treatments. DOC use was similar in all the + DOM treatments but was fastest in the intermediate DIN/DIP treatment (Treat#2; Fig S5 in S1 Appendix). Treat #2, also showed the fastest increase in [H^+^] (i.e., decrease in pH; Fig S6 in S1 Appendix), and was the treatment that showed the greatest nitrification-related decrease in NH_4_^+^ and increase in NO_3_^-^ (Fig 2). Control #2 also showed nitrification, while changes in NO_2_^-^ in Treat #3 suggest that that treatment was also entering conditions conducive to nitrification when the experiment stopped (Fig 2).

**Fig 3:**
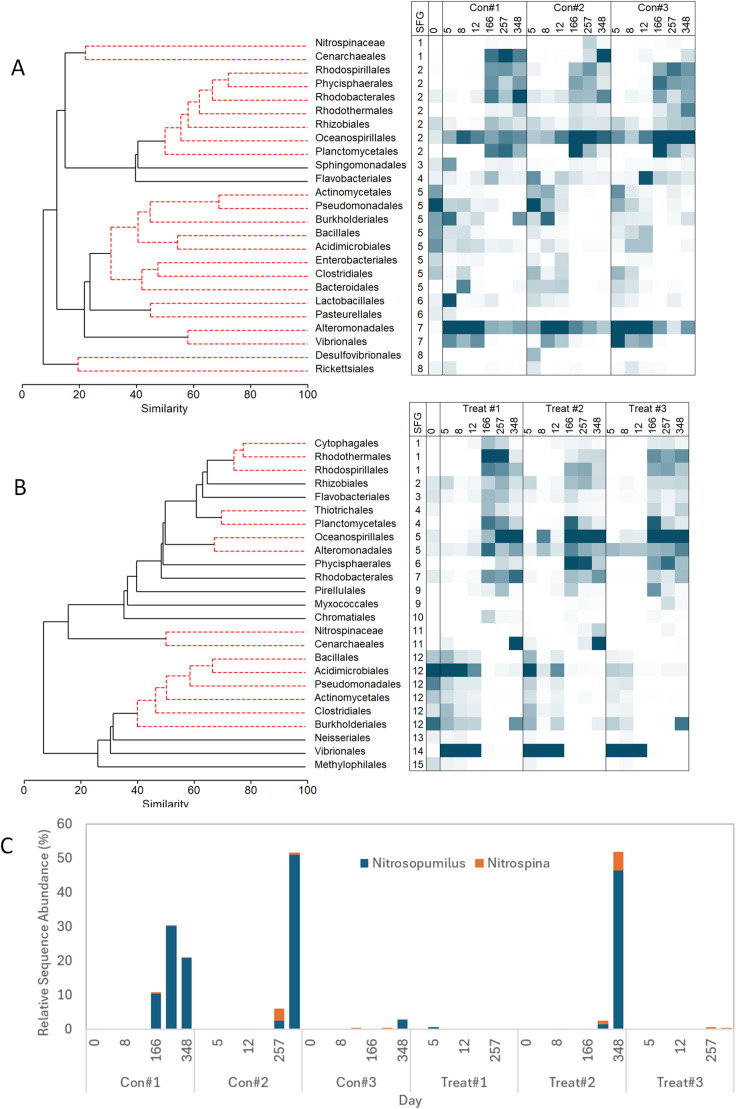
Comparison of changes to prokaryote community composition during Exp.II. These are shown within A) control and B) DOM-amended treatments containing 2.5, 15 or 75 µM NH_4_^+^. 16S rRNA gene sequence data were grouped into Orders and Classes, and their co-variance examined using a SIMRPOF test. Plots indicate which Orders and Classes were indistinguishable (SFG = SIMPROF group factor). Heatmaps indicating relative sequence abundance are shown alongside. The relative sequence abundance of *Nitrosopumilus* sp. and *Nitrospina* sp. are shown in detail in panel (C). The control and +DOM treatments each have different levels of NH_4_^+^ addition; see Tables 1 and S1 (S1 Appendix) for culture conditions.

Analyses to determine co-variance of dominant prokaryote orders highlights the effect of the addition of fresh DOM and NH_4_^+^ on the structure of the microbial community present. We noted marked differences in relative sequences abundances within the first 12 days compared to the later 166–257 days of the experiment (Fig 3 and Supplementary Table S2 and Fig S3 in S1 Appendix). Control treatments, receiving no DOM addition, were initially dominated with Alteromonadales (32.4% + /- 16.5% relative sequence abundance), Vibrionales (9.7% + /- 7.3%) and Oceanospirillales (9% + /- 4%) (Fig 3A) whereas the + DOM treatments were primarily dominated by Vibrionales (64% + /- 19%) (Fig 3B and Fig S3 in S1 Appendix).

Without the + DOM addition, the relative sequence abundances of Alteromonadales and Vibrionales were indistinguishable (SFG 7; Fig 3A). Within the first 12 days for control treatments, no discernable differences were detected within treatments receiving higher concentrations of NH_4_^+^ (Con#2 and Con#3; Fig 3A and Supplementary Table S2 and Fig S3 in S1 Appendix). In contrast, within the + DOM treatments, the microbial composition of ‘+DOM + 2.5 µM NH_4_^+^’ (Treat #1) differed from the treatments amended with 15 µM or 75 µM NH_4_^+^ (Treat #2 and Treat #3); the presence of added DOM thus affected the impact of higher inorganic nutrient concentrations on community composition.

Vibrionales dominated all + DOM treatments in the first 12 days (62% + /- 18%) but the relative sequence abundance of Acidimicrobiales (20.5% + /- 1.6%) was higher in Treat #1. There were no differences between Treat #2 and Treat #3, the community containing higher relative sequence abundance of SFG5 members Alteromonadales (6% + /- 3%) and Oceanospirillales (5% + /- 6%) (Fig 3B and Table S2 and Fig S3 in S1 Appendix).

The numbers of amplicon sequence variants (ASVs) that could be detected within all treatments increased over time (Fig S3A in S1 Appendix). From days 166–348, the effect of the initial fresh DOM addition on community structure was not as marked but was still discernible (Table S2 in S1 Appendix). For both the + DOM treatments and the controls, the microbial composition of vessels with no NH_4_^+^ addition differed from those bottles amended with 15 µM or 75 µM NH_4_^+^, but there were no differences in the community composition present within treatments amended with 15 µM versus 75 µM NH_4_^+^ (Table S2 in S1 Appendix). For the control treatments, this was due to subtle differences in the relative sequence abundance of SFG2 members (less Rhodospirillales and Planctomycetia but more Oceanospirillales) alongside Crenarchaeota (SFG1) (Fig 3A). From days 166–348, Treat #2 and Treat #3 contained higher relative sequence abundance of Oceanospirillales and Phycisphaerae, but less Alteromonadales (Fig 3B and Fig S3 in S1 Appendix). For the + DOM treatments, patterns of co-variance amongst the top 25 dominant Orders were more complex (Fig 3B and Fig S3 in S1 Appendix), with several Orders grouping differently when compared to the controls (e.g., Oceanospirillales co-varied with Alteromonadales).

The archaeal order Cenarchaeales grouped with the ammonia oxidizer Genus *Nitrosopumilus* sp. Together with the nitrite-oxidising bacterium *Nitrospina* sp., a high relative sequence abundance of nitrifying prokaryotes within both control and ‘+DOM + 15 µM NH_4_^+^’ on days 257 and 348 were apparent (Fig 3C). In both the control (SFG 1; Fig 3A) and the + DOM treatments (SFG 11; Fig 3B), the relative sequence abundance of Cenarchaeales and Nitrospinaceae significantly co-varied. For both the controls and ‘+DOM + 15 µM NH_4_^+^’ treatments, there was good correlation between the relative sequence abundance of *Nitrosopumilus* sp. and *Nitrospina* sp. and the decrease in NH_4_^+^ over time (control: R^2^ = 0.705; *p* < 0.001 and DOM: R^2^ = 0.701; *p* = 0.001) and the increase in NO_3_^-^ (control: R^2^ = 0.556; *p* < 0.001 and DOM: R^2^ = 0.649; *p* < 0.001). *Nitrosopumilus* sp. were also present within Con #1 on days 166, 257 and 348 (Fig 3C). Although there was good correlation between the increase in NO_3_^-^ and nitrifier relative sequence abundance within these treatments (R^2^ = 0.887; *p* < 0.001), there was no correlation between nitrifier relative sequence abundance and NH_4_^+^ concentrations (R^2^ = 0.015; *p* d= 0.588).

### Simulations

**Fig 4:**
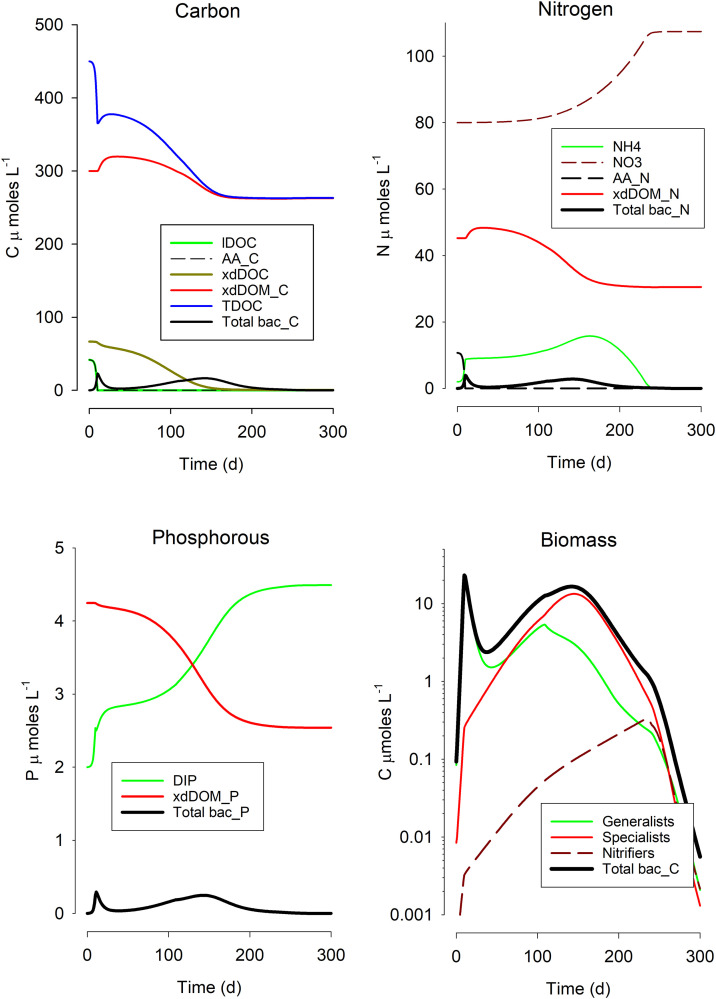
Simulated progression of the degradation of DOM and nitrification. Forms of DOM shown are: labile DOC (lDOC), amino and nucleic acids and allied small polymers (AA), polymeric DOC containing no N or P requiring extensive extracellular digestion (xdDOC), complex polymeric DOM containing N and/or P requiring extensive extracellular digestion (xdDOM). The microbial community was described as those capable of rapid growth using lDOC + AA but poorly able to use xdDOC + xdDOM (‘Generalists’ in the Biomass plot), ’Specialists’ capable of also exploiting the more complex xdDOC + xdDOM, and ‘Nitrifiers’ which were also capable of exploiting lDOC + AA. Ammonia/ammonium (NH4), nitrate (NO3) and dissolved inorganic phosphate (DIP) are also shown, together with total DOM-C (TDOC), total microbial biomass in terms of C, N, P (Tbac_C, Tbac_N, Tbac_P). See Fig S7 (S1 Appendix) for changes in DIC, oxygen, acidity and DOM C:N.

Simulations replicated the generality of the sequence of events seen in the experiments (Fig 4) but providing more insight regarding DOM fractions (not assessed empirically) and microbial functional type succession. The model simulated first the appearance of generalist exploiters of highly labile DOM, then the appearance of the specialists that were capable of exploiting more recalcitrant DOM, and then of the increased competitive advantage of the slower growing nitrifiers. The model describes bacteria/archaea community abundance in terms of C-biomass and therefore model simulations are not directly comparable with the numeric abundance shown in Figs 1 and 2 due to the significant differences in organism size (for example, *Alteromonas* is ca. 10x the cell size of *Nitrosopumulus*), and also because we have no measure of the proportions of the population that grew adhered to the culture bottles. However, the pattern of simulated progression is consistent with the observed changing bacteria/archaea communities (Fig 3), with increased abundance of nitrifiers toward the end of the experiment. Furthermore, the development of the simulated bacteria+archaea biomass is consistent with estimates of biomass in the experiments (calculations presented in the legend to Fig S5 in S1 Appendix). Simulated patterns of DOM-C, ammonium, nitrate and phosphate, which are consistent with those observed in the experiments, also provide a mechanistic explanation of the interplay between DOM and the microbial community present in the flasks. Thus, during the simulated degradation of DOM (with an increased proportion of xdDOM to the total in Fig 4), those generalist bacteria poorly equipped to degrade that recalcitrant DOM (‘generalists’ in Fig 4) still continued to grow, albeit at a slower rate as they were resource limited, using forms of DOM made available to them through the activity of the specialist DOM degraders (Fig 4). This labile DOM also supported the initial increase in the nitrifier population (Fig 4); when that support decreased sufficiently, the nitrifiers exploited the ammonium, leading to an increase in nitrate (Fig 4).

## Discussion

### Dynamics of DOM consumption and appearance of nitrification

In our experiments, we followed the consumption of microalgal-derived DOM by natural marine microbial communities over extended timeframes. The time course of events in these experiments, along with the analysis of simulations, suggest that the DOM pool was initially dominated by labile forms (which are rapidly consumed [2], with attendant nutrient regeneration) and then increasingly enriched in recalcitrant forms that becomes conducive to the emergence of communities of bacteria and archaea that support nitrification. The increasing prominence of nitrifiers occurs after many months of DOM degradation, when DOM concentration remains relatively stable (i.e., the DOM becomes functionally recalcitrant, Figs 1, 2 and 4), suggesting that the composition and concentration of DOM components was unable to support a good net growth rate of non-nitrifiers.

The implication of our results is that the appearance of nitrification signals a near-terminal point in the consumption of non-recalcitrant DOM sources by the Microbial Carbon Pump activities [3], marking the onset of carbon limitation for the heterotrophic bacteria community. These dynamics suggest that nitrification is not only dependent on processes promoting ammonium remineralization, as previously suggested [15,16,29], but also on the competition between nitrifiers and heterotrophic bacteria for readily usable sources of carbon and energy. When the labile DOM, generated both directly (through leaking and lysis) and indirectly (via grazing) by primary producers, is exhausted and the remaining DOM fractions become increasingly recalcitrant, the net growth of heterotrophic bacteria decreases (in many instances likely with their death) while that of the nitrifiers continues, supported by the oxidation of ammonium regenerated by the earlier activities upon labile DOM. We also noted a lack of correlation between nitrifier relative sequence abundance and NH_4_^+^ concentrations in our study. From this information, we hypothesize that changes in DOM composition, in terms of relative decrease of labile vs recalcitrant forms, is a key feature explaining the observed variability in the NH_4_^+^-normalized nitrification rate in the ocean [9]. This hypothesis is consistent with the findings that ammonia oxidation is considerably high in oligotrophic areas such as the subtropical gyres [29], where recalcitrant DOM accumulates [30]. Our results are also in line with previous findings suggesting that the addition of labile organic compounds suppresses nitrification in rivers by boosting the heterotrophic bacterial community at the expense of chemolithotrophs [14], these latter being less efficient in other resource acquisitions, such as of PO_4_^---^ [31].

Although chemical analyses (e.g., mass spectroscopy [32]) to confirm the transformation of DOM were not included in our studies, we argue that changes in DOM compositions, making the pool progressively enriched in recalcitrant fractions (as previously reported [33]), is the most plausible explanation for the observed stability of DOM after the first month of the experiment incubations. Additionally, manipulations of the model clearly showed that it is not possible to simulate the experimental results without considering the development of recalcitrance within the DOM pool (Fig 4). Furthermore, the model also simulates an increase in the C:N ratio within DOM during the experiment (Fig S7 in S1 Appendix); the progressive enrichment in carbon is one of the features indicative of DOM “aging” and elevated C:N ratios within organic matter (relative to the canonical Redfield ratio of 6.6 mol/mol) is commonly associated with recalcitrance [3,34]. It is important to stress here that while the setup of our model (i.e., length of the run, initial conditions, and treatments) was informed by the experiments, the model structure itself was based on literature knowledge about microbial physiology, ecology and biogeochemistry (S1 Appendix) and, therefore, is independent from the experiments. In other words, the simulated dynamic of DOC is an emergent property due to the combinations of existing information on DOC-bacteria interactions as interpreted by the model conceptual framework.

Simulations also suggest that the timing of the appearance of nitrification, as a clear transition from ammonium consumption to nitrate production (Fig S8 in S1 Appendix), is affected by the initial concentration of the different components of the bacteria community. The *in silico* experiment shown in Fig S8 (S1 Appendix) shows that if the initial abundance of nitrifiers (i.e., in the inoculum) is substantially higher than in the empirical experiments, then the competitive head-start in the growth of the heterotrophic bacteria is less marked; a noticeable amount of ammonia/ammonium was then immediately oxidized to nitrate and nitrifiers grow concomitantly with the heterotrophic counterpart (Fig S8 in S1 Appendix).

**Fig 5:**
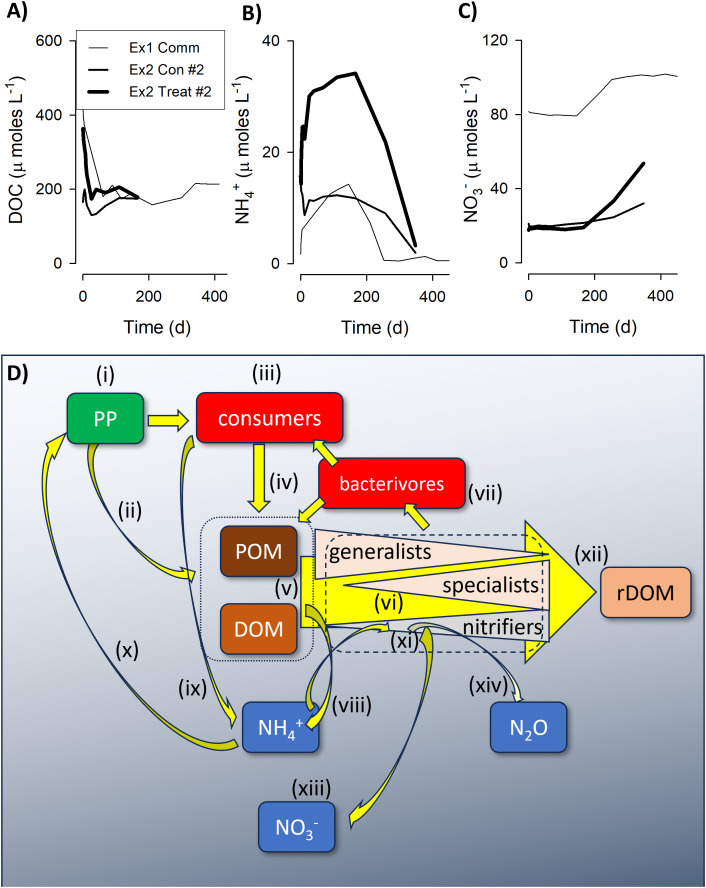
Suggested interactions between DOM production and decay in the ocean water column, with concurrent cycling of inorganic N. This schematic is informed from experimental and simulation results. Panels A), B) and C) show the DOC, NH_4_^+^ and NO_3_^-^ results from incubations in experiments 1 (Fig 1, ‘*Community*’) and 2 (Fig 2, ‘*Con #2*’, ‘*Treat #2*’) in which nitrification was seen to develop after a protracted period of DOM processing. Panel D) shows the proposed interactions, described in the text, and applied in a natural setting. Phototrophic production (i) releases DOM, with dead organisms contributing to POM (ii). Successions of consumers (iii) also produce DOM and POM (iv). Advection and mixing moves these materials and allied microbes to below the photic zone. Collective pools of DOM and POM (v), initially dominated by labile forms, are degraded by generalists and increasingly by specialist successions of bacteria and archaea (vi). Amongst these decomposers will reside populations that can contribute to nitrification as and when conditions allow. The total decomposer population (biomass and composition; dash-lined box) is also affected by grazing activity (vii). The collective activities of heterotrophic decomposers (viii) and grazers (ix) produce ammonium, which can support not only phototrophic production in the surface waters (x), but also the activities of nitrifiers (xi). The latter becomes increasingly likely as the POM + DOM pool is progressively denuded of more labile forms, leaving recalcitrant rDOM (xii). Nitrification produces nitrate (xiii), which can support phototrophic production. However, both the generic decomposer and consumer activities, and also nitrification, consume much oxygen; away from the surface waters where phototrophs produce oxygen, the action of nitrifiers under hypoxic conditions releases N_2_O (xiv), which is a potent green-house gas.

In Fig 5 we present a schematic pulling together the lines of evidence that emerge from our study. The observed dynamics of nitrifiers thus reflects a combination of their low initial abundance, in consequence of poor competitive advantage in the high-light waters from which the microbial community was sourced and where most DOM is released, and the deteriorating competitive advantage of heterotrophic bacteria as DOM was degraded. In this system, nitrification only becomes apparent when the collective heterotrophic bacteria activity (i.e., as the product of C-specific growth and biomass) is lower than (Figs 1 and 2), or comparable with (Fig S8 in S1 Appendix), that of nitrifiers. We have shown that the abundance of the nitrifier population relative to that of the heterotrophic bacteria, coupled with the carbon limitation that develops as DOM is degraded, play a role in the competition between heterotrophic bacteria and nitrifiers. Furthermore, the evidence that the same DOM pool may appear as functionally recalcitrant for specific bacteria (in this case, *Alteromonas*, Figs 1 and S2 in S1 Appendix) suggests that also the quality (rather than the total amount) of the heterotrophic bacteria community can contribute to the observed variability of nitrification in the ocean.

### Community dynamics

Microbial community data from Exp II shows that initially, the community was dominated by fast-growing generalists, many of which were identified in a similar study tracking the degradation organic matter [35,36] including Gammaproteobacteria (in this study, specifically Alteromonadales, Oceanospirillales, Pseudomonadaes, Vibrionales), Acidimicrobiia and Burkholderiales. As DOM becomes increasingly recalcitrant, it is expected that specialists adapted to exploit low-energy substrates would begin to emerge; these are expected to be Bacteroidetes and Firmicutes but studies of a similar length to that presented here suggest a wider diversity of microbes are present with microbes including Alphaproteobacteria, Gammaproteobacteria, Planctomycetes and Thaumarchaeota dominating when the DOM is expected to be increasingly recalcitrant [36,37]. Similar to earlier observations [37], this was accompanied by an increase to microbial diversity over time, presumably related to the greater complexity of DOM present.

The complexity of the microbial community (Fig 3) is consistent with the simulation output (Fig 4), where generalists (e.g., many Proteobacteria) continue to survive in conditions dominated by increasing recalcitrant DOM by exploiting residual concentrations of labile DOM cleaved by extracellular digestion from less-labile DOM through the activity of specialists (e.g., members of the Bacteroidetes) and also from the lysis of dying microbes. For example, Alteromonadales were present even in bottles after long incubations (Fig 3), in which most DOM would not have been labile, while *Alteromonas* itself is incapable of readily exploiting such material [18]. There is no indication that the energy gain from nitrification helps to further process DOM, and indeed the liberation of labile DOM with nitrifier autotrophy [12] would provide forms of more labile DOM that may repress the need by heterotrophs to exploit recalcitrant DOM. Where sufficient labile DOM is available, the nitrifiers may either not express high rates of nitrification, or they may simply be outgrown, or outcompeted, by other bacteria and archaea.

### Potential mechanisms restricting or inhibiting nitrification

While the mechanism proposed above explains the patterns observed in several of our experimental setups (summarized in Fig 5A–C), a clear nitrification signal is absent in certain treatments of Exp.II, notably treat#1, con#3 and treat #3 (con#1 was not expected to display nitrification due to lack of DOM to support nutrient regeneration and the absence of additional NH_4_^+^ concentration). This indicates that specific environmental factors may constrain the above-described dynamics, which warrants further investigation. However, based on the different biogeochemical features between experiments where nitrification was apparent, versus those where not (i.e., treat#1, con#3 and treat #3), we can offer some explanations of possible mechanisms suppressing nitrification. For example, in treat #1, nitrifiers could have been NH_4_^+^ limited in the first part of the experiment and then, in the second part, top-down controlled by small grazers. The grazer population peaks at around day 70, later in comparison with the other experiments where nitrification is apparent (Fig S4 in S1 Appendix). Nitrification does not appear also in the high-ammonium bottles, Con#3 and Treat#3 (Fig 2), although there are some signs of the emergence of nitrifiers towards the end of those incubations (Figs 3 and S3 in S1 Appendix). We suggest that in these bottles the growth of nitrifiers was inhibited by the relatively high concentrations of ammonia/ammonium. This is consistent with reports from freshwater ecology [38–41] indicating that such conditions can conspire to promote niche separations between comammox and other nitrifiers. Although 75 µM ammonium is not high relative to levels in freshwater, sediment and wastewater systems, it is well above the concentration observed in the waters where the inocula were obtained (Western English Channel) where NH_4_^+^ is consistently below 1 µM [42], as it is in oceanic waters in general.

Away from areas of net oxygen production and gas exchange, nitrification would also be limited by oxygen availability. Our simulations show that oxygen limitation became increasingly likely toward the end of the experiments when the bulk DOM was seen to become C-enriched (Fig S7 in S1 Appendix). However, due to low oxygen availability, the C:N ratio did not increase to the levels expected after prolonged microbial processing [33]. The proposed interplay between DOM degradation and the relative increase in nitrifying prokaryotes might also enhance the production of the powerful greenhouse gas N_2_O [43–45] (Fig 5D). N_2_O is mainly released as a byproduct during nitrification (other than with denitrification), and is enhanced by hypoxia [46], a condition that develops during the events we noted in our simulations (Fig S7 in S1 Appendix).

## Conclusion

Our work describes an interplay between DOM processing, microbial community succession and nitrification with dynamics operating over extended time scales, and thence in nature over extended spatial scales as water masses flow, are advected and mixed. Within the limits imposed by our experimental conditions (e.g., lack of full planktonic components, DOM sourced from a single phytoplankton type) our results highlight potentially important implications for future ocean biogeochemistry impacted by climate change. Climate models predict a global decrease in ocean primary production due to increased water column stratification and the consequent oligotrophication of oceanic sunlit waters [47]. Nutrient-stressed phototrophic communities have been suggested to produce more recalcitrant (carbon-rich) forms of DOM with a potential impact on the microbial community and an enhancement of the MCP [48]. The relative increase in recalcitrant fractions within the bulk DOM pool could therefore limit the growth and fitness of the heterotrophic bacteria community leaving more opportunities for chemolithotrophic activity through nitrification in future oceans. However, the decreased concentration of nutrient-N in those surface waters would alter the C:N of the DOM and thus the production of ammonium required by nitrifiers. Even so, the above-described mechanisms would imply a relative increase in CO_2_ fixation over CO_2_ production, providing negative feedback to climate change. This flags the importance for future studies synergistically exploring DOM processing, nitrification and also N_2_O production under different conditions (quality and quantity) of DOM supply.

## Supporting information

S1 Appendix Supplementary Methods and Results. (PDF)

S2 Dataset Experimental data. (XLSX)

## References

[1] Hansell DA, Orellana MV. Dissolved Organic Matter in the Global Ocean: A Primer. Gels. 2021;7(3):128. doi: 10.3390/gels7030128 34563014 PMC8482078

[2] Hach PF, Marchant HK, Krupke A, Riedel T, Meier DV, Lavik G, et al. Rapid microbial diversification of dissolved organic matter in oceanic surface waters leads to carbon sequestration. Sci Rep. 2020;10(1). doi: 10.1038/s41598-020-69930-y PMC7400608 32747679

[3] Jiao N, Herndl GJ, Hansell DA, Benner R, Kattner G, Wilhelm SW, et al. The microbial carbon pump and the oceanic recalcitrant dissolved organic matter pool. Nat Rev Microbiol. 2011;9(7):555–555. doi: 10.1038/nrmicro2386-c5 20601964

[4] Benner R, Amon RMW. The Size-Reactivity Continuum of Major Bioelements in the Ocean. Annu Rev Mar Sci. 2015;7(1):185–205. doi: 10.1146/annurev-marine-010213-135126 25062478

[5] Ward BB. Nitrification In Earth Systems and Environmental Sciences. Elsevier; 2013. doi: 10.1016/B978-0-12-409548-9.00697-7

[6] Wright CL, Lehtovirta-Morley LE. Nitrification and beyond: metabolic versatility of ammonia oxidising archaea. The ISME Journal. 2023;17(9):1358–68. doi: 10.1038/s41396-023-01467-0 37452095 PMC10432482

[7] Santoro AE, Richter RA, Dupont CL. Planktonic marine archaea. Annu Rev Mar Sci. 2019;11:131–58. 10.1146/annurev-marine-121916-063141 30212260

[8] Palomo A, Dechesne A, Pedersen AG, Smets BF. Genomic profiling of Nitrospira species reveals ecological success of comammox Nitrospira. Microbiome. 2022;10(1):204. 36451244 10.1186/s40168-022-01411-y PMC9714041

[9] Yool A, Martin AP, Fernández C, Clark DR. The significance of nitrification for oceanic new production. Nature. 2007;447(7147):999–1002. doi: 10.1038/nature05885 17581584

[10] Pajares S, Ramos R. Processes and Microorganisms Involved in the Marine Nitrogen Cycle: Knowledge and Gaps. Front Mar Sci. 2019;6. doi: 10.3389/fmars.2019.00739

[11] Clark DR, Rees AP, Ferrera C, Al-Moosawi L, Somerfield PJ, Harris C. Nitrification in the oligotrophic Atlantic Ocean. Biogeosciences Discussions. 2021:1–29.

[12] Zehr JP, Ward BB. Nitrogen Cycling in the Ocean: New Perspectives on Processes and Paradigms. Appl Environ Microbiol. 2002;68(3):1015–24. doi: 10.1128/aem.68.3.1015-1024.2002 11872445 PMC123768

[13] Bayer B, McBeain K, Carlson CA, Santoro AE. Carbon content, carbon fixation yield and dissolved organic carbon release from diverse marine nitrifiers. Limnology & Oceanography. 2022;68(1):84–96. doi: 10.1002/lno.12252 37064272 PMC10092583

[14] Butturini A, Battin TJ, Sabater F. Nitrification in stream sediment biofilms: the role of ammonium concentration and DOC quality. Water Research. 2000;34(2):629–39.

[15] Santoro AE, Saito MA, Goepfert TJ, Lamborg CH, Dupont CL, DiTullio GR. Thaumarchaeal ecotype distributions across the equatorial Pacific Ocean and their potential roles in nitrification and sinking flux attenuation. Limnology & Oceanography. 2017;62(5):1984–2003. doi: 10.1002/lno.10547

[16] Zakem EJ, Al-Haj A, Church MJ, van Dijken GL, Dutkiewicz S, Foster SQ, et al. Ecological control of nitrite in the upper ocean. Nat Commun. 2018;9(1). doi: 10.1038/s41467-018-03553-w PMC5865239 29572474

[17] Carlson CA, Hansell DA. DOM sources, sinks, reactivity, and budgets. Biogeochemistry of marine dissolved organic matter. 2015:65–126.

[18] Polimene L, Clark D, Kimmance S, McCormack P. A substantial fraction of phytoplankton-derived DON is resistant to degradation by a metabolically versatile, widely distributed marine bacterium. PLoS ONE. 2017;12(2):e0171391. doi: 10.1371/journal.pone.0171391 PMC5291467 28158278

[19] Halewood E, Opalk K, Custals L, Carey M, Hansell DA, Carlson CA. Determination of dissolved organic carbon and total dissolved nitrogen in seawater using High Temperature Combustion Analysis. Front Mar Sci. 2022;9. doi: 10.3389/fmars.2022.1061646

[20] Tarran GA, Bruun JT. Nanoplankton and picoplankton in the Western English Channel: abundance and seasonality from 2007–2013. Progress in Oceanography. 2015;137:446–55. doi: 10.1016/j.pocean.2015.04.024

[21] Parada AE, Needham DM, Fuhrman JA. Every base matters: assessing small subunit rRNA primers for marine microbiomes with mock communities, time series and global field samples. Environmental Microbiology. 2015;18(5):1403–14. doi: 10.1111/1462-2920.13023 26271760

[22] Apprill A, McNally S, Parsons R, Weber L. Minor revision to V4 region SSU rRNA 806R gene primer greatly increases detection of SAR11 bacterioplankton. Aquat Microb Ecol. 2015;75(2):129–37. doi: 10.3354/ame01753

[23] Bolyen E, Rideout JR, Dillon MR, Bokulich NA, Abnet CC, Al-Ghalith GA, et al. Reproducible, interactive, scalable and extensible microbiome data science using QIIME 2. Nat Biotechnol. 2019;37(8):852–7. doi: 10.1038/s41587-019-0209-9 31341288 PMC7015180

[24] Callahan BJ, McMurdie PJ, Rosen MJ, Han AW, Johnson AJA, Holmes SP. DADA2: High-resolution sample inference from Illumina amplicon data. Nat Methods. 2016;13(7):581–3. doi: 10.1038/nmeth.3869 27214047 PMC4927377

[25] McDonald D, Price M, Goodrich J, Nawrocki EP, DeSantis TZ, Probst A. An improved Greengenes taxonomy with explicit ranks for ecological and evolutionary analyses of bacteria and archaea. ISME J. 2012;6:610–8. 22134646 10.1038/ismej.2011.139 PMC3280142

[26] Clarke KR, Gorley RN. PRIMER v6: User Manual/Tutorial. PRIMER-E, Plymouth. 2006.

[27] Somerfield PJ, Clarke KR. Inverse analysis in non-parametric multivariate analyses: distinguishing groups of associated species which covary coherently across samples. Journal of Experimental Marine Biology and Ecology. 2013;449:261–73. doi: 10.1016/j.jembe.2013.10.002

[28] Flynn KJ, Mitra A. DRAMA - a cybernetic approach for Plankton Digital Twins. Zenodo. 2023. doi: 10.5281/zenodo.7848329

[29] Shiozaki T, Ijichi M, Isobe K, Hashihama F, Nakamura K, Ehama M, et al. Nitrification and its influence on biogeochemical cycles from the equatorial Pacific to the Arctic Ocean. The ISME Journal. 2016;10(9):2184–97. doi: 10.1038/ismej.2016.18 26918664 PMC4989309

[30] Hansell DA. Recalcitrant Dissolved Organic Carbon Fractions. Annu Rev Mar Sci. 2013;5(1):421–45. doi: 10.1146/annurev-marine-120710-100757 22881353

[31] de Vet WWJM, van Loosdrecht MCM, Rietveld LC. Phosphorus limitation in nitrifying groundwater filters. Water Research. 2012;46(4):1061–9. doi: 10.1016/j.watres.2011.11.075 22209259

[32] Hertkorn N, Benner R, Frommberger M, Schmitt-Kopplin P, Witt M, Kaiser K, et al. Characterization of a major refractory component of marine dissolved organic matter. Geochimica et Cosmochimica Acta. 2006;70(12):2990–3010. doi: 10.1016/j.gca.2006.03.021

[33] Ogawa H, Amagai Y, Koike I, Kaiser K, Benner R. Production of Refractory Dissolved Organic Matter by Bacteria. Science. 2001;292(5518):917–20. doi: 10.1126/science.1057627 11340202

[34] Osterholz H, Niggemann J, Giebel H-A, Simon M, Dittmar T. Inefficient microbial production of refractory dissolved organic matter in the ocean. Nat Commun. 2015;6(1). doi: 10.1038/ncomms8422 26084883

[35] Pontiller B, Martínez-García S, Lundin D, Pinhassi J. Labile dissolved organic matter compound characteristics select for divergence in marine bacterial activity and transcription. Frontiers in Microbiology. 2020;11:588778. 33101262 10.3389/fmicb.2020.588778 PMC7546218

[36] Wang Y, Xie R, Shen Y, Cai R, He C, Chen Q, et al. Linking Microbial Population Succession and DOM Molecular Changes in Synechococcus -Derived Organic Matter Addition Incubation. Microbiol Spectr. 2022;10(2). doi: 10.1128/spectrum.02308-21 PMC9045170 35380472

[37] LaBrie R, Péquin B, Fortin St-Gelais N, Yashayaev I, Cherrier J, Gélinas Y, et al. Deep ocean microbial communities produce more stable dissolved organic matter through the succession of rare prokaryotes. Sci Adv. 2022;8(27). doi: 10.1126/sciadv.abn0035 PMC11323801 35857452

[38] Smith RV, Burns LC, Doyle RM, Lennox SD, Kelso BHL, Foy RH, et al. Free ammonia inhibition of nitrification in river sediments leading to nitrite accumulation. American Society of Agronomy, Crop Science Society of America, and Soil Science Society of America; 1997;Vol. 26, No. 4, p. 1049–55.

[39] Paśmionka IB, Bulski K, Herbut P, Boligłowa E, Vieira FMC, Bonassa G, et al. Toxic Effect of Ammonium Nitrogen on the Nitrification Process and Acclimatisation of Nitrifying Bacteria to High Concentrations of NH4-N in Wastewater. Energies. 2021;14(17):5329. doi: 10.3390/en14175329

[40] Cui L, Li D, Wu Z, Xue Y, Song Y, Xiao F, et al. Effects of Nitrification Inhibitors on Nitrogen Dynamics and Ammonia Oxidizers in Three Black Agricultural Soils. Agronomy. 2022;12(2):294. doi: 10.3390/agronomy12020294

[41] Yang X, Yu X, He Q, Deng T, Guan X, Lian Y, et al. Niche differentiation among comammox (Nitrospira inopinata) and other metabolically distinct nitrifiers. Front Microbiol. 2022;13. doi: 10.3389/fmicb.2022.956860 PMC9515657 36187961

[42] McEvoy AJ, Atkinson A, Airs RL, Brittain R, Brown I, Fileman ES, et al. The Western Channel Observatory: a century of physical, chemical and biological data compiled from pelagic and benthic habitats in the western English Channel. Earth Syst Sci Data. 2023;15(12):5701–37. doi: 10.5194/essd-15-5701-2023

[43] Zamora LM, Oschlies A. Surface nitrification: A major uncertainty in marine N2O emissions. Geophysical Research Letters. 2014;41(12):4247–53. doi: 10.1002/2014gl060556

[44] Ji Q, Buitenhuis E, Suntharalingam P, Sarmiento JL, Ward BB. Global Nitrous Oxide Production Determined by Oxygen Sensitivity of Nitrification and Denitrification. Global Biogeochemical Cycles. 2018;32(12):1790–802. doi: 10.1029/2018gb005887

[45] Breider F, Yoshikawa C, Makabe A, Toyoda S, Wakita M, Matsui Y, et al. Response of N2O production rate to ocean acidification in the western North Pacific. Nat Clim Chang. 2019;9(12):954–8. doi: 10.1038/s41558-019-0605-7 31857827 PMC6923134

[46] Wan XS, Hou L, Kao S-J, Zhang Y, Sheng H-X, Shen H, et al. Pathways of N2O production by marine ammonia-oxidizing archaea determined from dual-isotope labeling. Proc Natl Acad Sci USA. 2023;120(11). doi: 10.1073/pnas.2220697120 PMC10243131 36888658

[47] Leonelli FE, Bellacicco M, Pitarch J, Organelli E, Buongiorno Nardelli B, de Toma V, et al. Ultra-oligotrophic waters expansion in the North Atlantic subtropical gyre revealed by 21 years of satellite observations. Geophysical Research Letters. 2022;49(21):e2021GL096965. doi: 10.1029/2021GL096965

[48] Polimene L, Sailley S, Clark D, Mitra A, Allen JI. Biological or microbial carbon pump? The role of phytoplankton stoichiometry in ocean carbon sequestration. Journal of Plankton Research. 2017;39(2):180–6.

