# Supplementary material for: The emergence of nitrification during DOM processing by marine microbial assemblages
Source: PLoS One. 2025 Dec 3;20(12):e0336919. doi: 10.1371/journal.pone.0336919 (PMC12674553; doi:10.1371/journal.pone.0336919)
Supplement: S1 Appendix — (PDF) [file pone.0336919.s001.pdf]

SUPPLEMENTARY INFORMATION

The emergence of nitrification during DOM processing by marine microbial assemblages

Flynn KJ[1], Clark DR[1,2], Tait K[1], Kimmance SA[1,3], Fileman ES[1], Polimene L[1,4]*

1) Plymouth Marine Laboratory, Prospect Place, Plymouth PL1 3DH, UK
2) Somerset Scientific Services, Unit 2a, Westpark, 26 Chelston, Wellington TA21 9AD, UK
3) College of Life and Environmental Sciences, University of Exeter, Stocker Road, Exeter, EX4 4QD, UK
4) European Commission, Joint Research Centre (JRC), Ispra, Italy

Supplementary Methods

Simulations

The following provides an overview of the bacteria model.

The bacterial model itself was largely developed as a private venture by KJ Flynn and ARK dynamics Ltd.

Incorporation of the nitrification component, configuration and deployment of the bacterial model for this project, were funded by UKRI-NERC through project NE/R011087/1.

A file for running the model, as used for this study, is available running under Powersim Studio 10.

Model Overview

The following describes a modelling structure to simulate the growth of marine aerobic bacteria as it exploits resources of different availabilities and labilities. The bacteria model comprises 4 state variables, 2 for C, plus N and P, all with units of mass m^{-3}. The model employs a coarse-grain systems biology approach, with exploitation of resources regulated (as in reality) through stress-related (de)repression. The model could (with the exception of nitrification) be used to describe growth of individual species; as described here, it is best suited to describe a bacterial functional type or a bacteria succession assemblage.

The model is based on the DRAMA (**D**ynamic **R**esource **A**ssimilation with **M**odulated **A**cquisition) conceptual framework (Flynn & Mitra 2023). The key feature of DRAMA is the use of multiple feedbacks from the C, N and P status of the modelled organism to modulate resource acquisitions and behaviour. DRAMA builds from well-established quota-style modelling concepts, but enhances them by inclusion of a C-metabolite quota. It

provides a comprehensive yet computationally tractable approach to describe organism growth exploiting multiple resource types.

Here, for a description of the activity and growth of bacteria, with reference to **Fig.MS1.1**, growth is a function of the physiological status with respect to C (CNu), N (NCu) and P (PCu). In the absence of sufficient inflow of resources from readily transportable forms (processes I – v), the ability to exploit other resources is enabled through a progressive de-repression ('switching-on') of mechanisms (**Fig.MS1.2**). These include the extracellular digestions ('xd' prefix) of polymeric DOC such as starch and fatty acids (xdDOC), of other polymeric molecules including proteins and cellulose (xdDOM_C xdDOM_N), and of xdDOM-P.

The ease of digestion of xdDOM_C and xdDOM_N is related to the N:C ratio of that DOM; the ease of digestion decreases as N:C decreases. The model thus uses a continuum recalcitrant-DOM argument, although the conceptual structure could equally well be configured for alternative strategies. The continuum argument assumes that all forms of DOM are usable at extremely high concentration irrespective of how poor them are nutritionally.

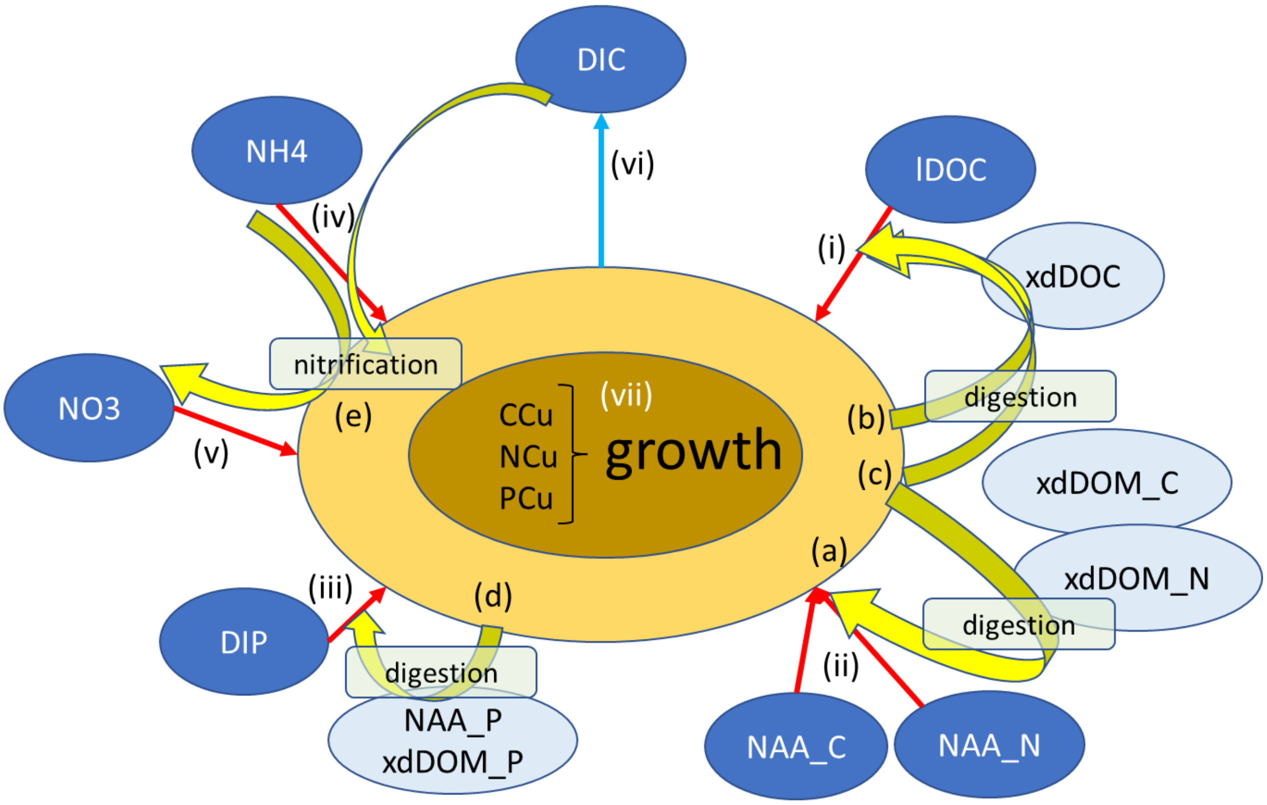

Fig.MS1.1 Schematic overview of the prokaryote model. Note the flows of material suitable for transport following extracellular digestion of polymeric forms of DOM (xdDOM). Growth is enabled via osmotrophy (red arrows from organic substrates) bringing in labile DOC (primary metabolites such as sugars—- lDOC; i), nucleic/amino acids (NAA_C + NAA_N, ii), as well as the inorganic nutrients DIP (iii), DIN as ammonium (NH4, iv) and nitrate (NO3, v). Respiration releases CO_2 (DIC, vi); respiration is increased in N-stressed cells, and even more so in P-stressed cells. Chemicals that require extracellular digestion (a-d) before transportation are xdDOC – polymeric DOC converted that requires to yield lDOC; xdDOM_C/N—- polymeric DOM forms of C and N converted to yield NAA_C/N; NAA_C/N— nucleic/amino acids, nucleic acids etc.; NAA_P and xdDOM_P—- polymeric DOM forms of P converted to yield DIP. If enabled, nitrification provides C-fixation at the expense of NH4, and produces NO3. See also **Table MS1.1**.

Table MS1.1 DOM chemical functional types for state variables in models for labile and poly-labile forms. Some inorganic forms are also listed as these are important resources for bacterial growth. **Labile** forms are likely all used by C-replete bacteria, and costs of bacterial usage are typically limited to standard anabolic respiration (including nitrate reduction, if appropriate). **Poly-labile** forms are used by nutrient-sufficient to nutrient-deplete bacteria, but not by nutrient-replete bacteria; i.e. usage is repressed by the ready assimilation rates of labile forms. Costs of bacterial usage not only include the standard anabolic costs, but also a cost for exoenzyme production in terms of C,N which is itself leaked to (added to) the poly-labile pool. Poly-labile P is recovered using alkaline phosphatase and nucleotidase expressed through relief of repression control if DIP is low and thus cellular P:C is depressed.

Lability	State Variable Name	Unit	Description	Mode of exploitation
Labile	**lDOC**	mgC m^{-3}	Sugars	direct uptake modulated by physiological status
	**NAA_C**	mgC m^{-3}	free amino acids and nucleic acids, assuming a fixed (average) N:C	direct uptake modulated by physiological status so growth using this source is by priority over usage of DIN
	**NH4**	mgN m^{-3}	Ammonium	direct uptake modulated by physiological status
	**NO3**	mgN m^{-3}	Nitrate	direct uptake modulated by physiological status under (de)repression chain so NH4 is used by priority
	**DIP**	mgP m^{-3}	Phosphate	direct uptake modulated by physiological status
Poly-labile	**xdDOC**	mgC m^{-3}	FA, polysaccharides	direct uptake following extracellular digestion
	**xdDOM_C**	mgC m^{-3}	proteins, NA etc assuming a fixed (average) N:C	direct uptake following extracellular digestion
	**xdDOP**	mgP m^{-3}	phosphorylated primary and secondary metabolites	direct uptake of P following extracellular digestion with the remaining C assumed remaining in the xdDOC pool

Nitrification may be enabled, if appropriate, when the C-status of archaea is sufficiently low (**Fig. MS1.2**). This is described as a simplified single-step process that in reality may be performed as two steps by different bacteria (Ward 2013; Bayer et al. 2023; Wright & Lehtovirta-Morley, 2023). Through chemolithoautotrophy, the energy gained from the oxidation of NH_4^+ to NO_3^- is used to enable CO_2-fixation, potentially also supporting mixotrophy.

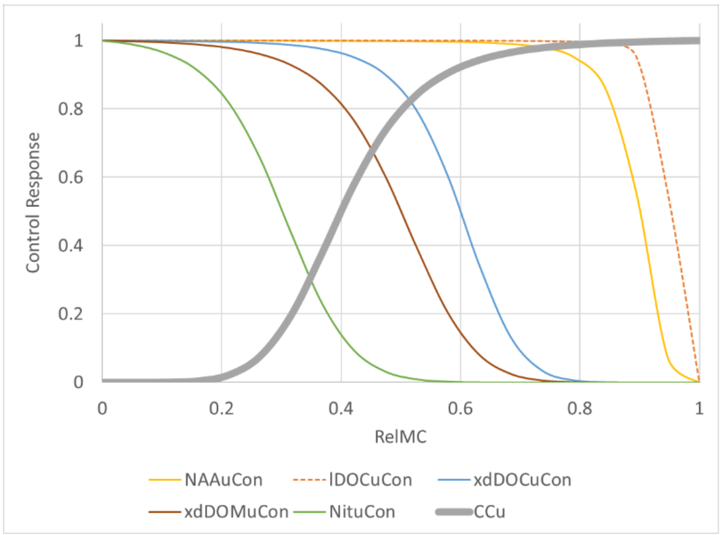

Fig.MS1.2 The DRAMA concept exploits changes in the nutritional state of the cell to modulate the potential to use different resources. This is performed using sigmoidal curve functions, which are themselves consistent with biochemical allosteric regulation. Here, the availability of metabolite C (RelMC) controls the use of different C-sources. As shown here, stress derepresses the ability to use labile DOC (lDOMuCon), nucleic/amino acids (NAAuCon), extracellularly digested DOC (xdDOCuCon), extracellular digested DOM (xdDOMuCon), and nitrification (NituCon). Ccu describes the concurrent C-status of the cell, which together with the N and P status, control growth. Note that the use of xdDOC, xdDOM and especially nitrification are incapable of supporting high growth rates as their de-repression aligns with low RelMC and hence low Ccu. These matters are discussed in detail in subsequent sections.

The model does not assume that bacteria themselves contribute to the production of a pool labelled as rDOM (recalcitrant or refractory xdDOM). On death the bacterial biomass is passed to xdDOM, the C:N:P stoichiometry of that material is good, such that it is readily exploited.

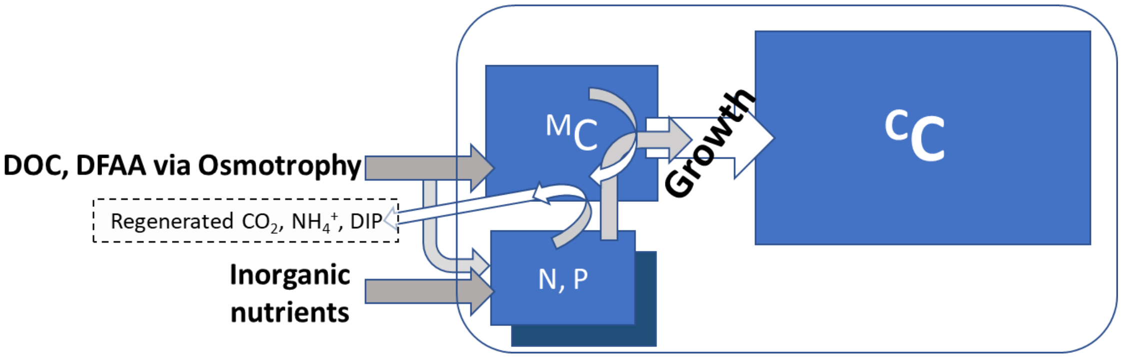

Fig.MS1.3 Schematic of the Bacteria-DRAMA model. Sources of C enter the metabolite ^MC pool, with any N or P associated with those organic molecules (plus inorganic N and P) entering the N or P pools. The structural C of the organism is in ^CC; ^MC + ^CC describes the total C content of the organism. Feedbacks from the C-quota (^MC:C), N-quota (N:C) or P-quota (P:C) are used to control acquisition of different nutrient sources. The values of these quotas collectively control the growth rate, as the movement of C from ^MC into ^CC.

Functional Equations

The following describes a C,N,P -based model. Functional-equations give a description of the model as text strings, with the form:

result = *f*{comma delimited list of factors involved in deriving the result}

Underlined terms in the equations are rates. Those in **bold** donate terms that provide a positive interaction (i.e., the result increases when the term increases; these are usually enacted via a curvi-linear function); terms not in bold may involve negative or more complex interactions (such as bell-shaped functions).

The equations are provided working backwards from the emergent organism growth rate, with descriptions of the steps enabling that rate to be attained.

State variables

The model comprises the following state variables and associated flows:

Core structure: bac^{C}C; mgC m^{-3}

d bac^{C}C/dt = {anabolism} – {catabolism when ^{M}C:^{T}C is critically low}

Metabolic pool of C: bac^{M}C ; mgC m^{-3}

d bac^{M}C/dt = {osmotrophy(C)} + {chemoautotrophy(C)} – {anabolism} – {catabolism} – {NO3-assimilation & reductant} – {DOM(C)-leak}

Organism-P : bacP ; mgP m^{-3}

d bacP/dt = {osmotrophy(P)} + {DIP-assimilation} – {P-regeneration} – {DOM(P)-leak}

Organism-N : bacN; mgN m^{-3}

d bacN/dt = {osmotrophy(N)} + {NH4-assimilation} + {NO3-assimilation} – {N-regeneration} – {DOM(N)-leak}

NOTE: In what follows, for brevity, state variable names in the equations do not include the prefix ‘bac’.

Total bacteria biomass C is given as, ^{T}C = ^{M}C+ ^{C}C.

Growth and nutrient status

Ultimately growth is a function of the nutritional status of the organism (in terms of elements C, N and P) and the maximum growth rate potential. The latter varies with temperature, T, around the value of μ_{max} at a reference temperature, μ_{maxRT}.

Growth = *f*{**C-status**, **N-status**, **P-status**, **μ_{max}**, losses}

μ_{max} = *f*{**μ_{maxRT}**, **T**}

The nutrient status defines the health of the organism in terms of C (^{M}C:^{T}C), N (N:^{T}C) and P (P:^{T}C), and is a function of various inputs and outputs. Inputs are associated with the use of dissolved organic substrates via osmotrophy and the use of inorganics, including via chemoautotropjhy (nitrification). Losses occur through respiration and regeneration, and also through the leakage of metabolites as dissolved organic matter (DOM), some of which may be recovered via osmotrophy. On death, bacterial biomass contributes to the xdDOM pool and is made available to the remaining bacteria.

C-status = *f*{**lDOC-uptake**, **xdDOC-assimilation**, **xdDOM_C-digestion**, **chemoautotrophy**, C-respiration, DOM-leak}

N-status = *f*{**NAA-uptake**, **DIN-uptake**, **xdDOM_N-digestion**, N-regeneration, AA-leak }

P-status = *f*{**DIP assimilation**, **NAA_P & xdDOM_P-digestion**, P-regeneration, DOM-leak}

Losses = *f*{**C-respiration**, **N-regeneration**, **P-regeneration**, **DOM-leak**}

Growth is associated with catabolic (including basal) and anabolic respiration. Anabolic respiration is affected by the flows of resources via the different mechanisms. Nitrate assimilation incurs an additional cost for reduction of nitrate to nitrite to ammonium. There are also losses of C, N, P required to preserve organism stoichiometry within the bounds of acceptable cellular C:N:P.

C-respiration = f{**μ_{max}**, **basal respiration**, **bacterial-C:N:P**, **C-assimilation**, **NO3-assimilation**}

C-assimilation = f{**osmotrophy**, **chemoautotrophy**}

N-regeneration = f{**C-respiration**, **bacterial-C:N**}

P-regeneration = f{**C-respiration**, **bacterial-C:P** }

DOM-leak is closely associated with osmotrophy (see further below).

Osmotrophy

Osmotrophy depends on the concentration of the substrates, [NAA], [lDOC], and the uptake kinetics parameters of the maximum uptake rate (V_{max}) and the substrate affinity (i.e., V_{max}/K). The uptake kinetics depend on the nutrient status of the organism; cells that are nutrient-stressed have a higher uptake potential and a high affinity.

lDOC-uptake = f\{C-status, **[lDOC]**, $\underline{^{lDOC}\mathbf{V}_{\mathbf{max}}}$, $\underline{^{lDOC}\mathbf{V}_{\mathbf{max}}}$**/**$\mathbf{K}_{\mathbf{lDOC}}$\}

$\underline{^{lDOC}V_{max}}$ = f\{N:C-status, $\underline{\boldsymbol{\mu}_{\mathbf{max}}}$\}

$1/K_{lDOC}$ = f\{N:C-status\}

NAA-uptake = f\{N:C-status, **[NAA]**, $\underline{^{AA}\mathbf{V}_{\mathbf{max}}}$, $\underline{^{AA}\mathbf{V}_{\mathbf{max}}}$**/**$\mathbf{K}_{\mathbf{AA}}$, \}

$\underline{^{AA}V_{max}}$ = f\{C-status, N-status, $\underline{\boldsymbol{\mu}_{\mathbf{max}}}$\}

$1/K_{NAA}$ = f\{**C-status**, **N-status**\}

Against the gains from osmotrophy there are losses with the leakage of lDOC and NAA. At especially high growth rates, which require a high nutrient status and hence a replete internal metabolite pool containing mM concentrations, material inevitably leaks. Osmotrophy may recover some of that leakage. The net leakage of N-containing DOM (as amino acids) is most significant during N-replete growth conditions, while leakage of lDOC (sugars) occurs especially with high ^{M}C (high C-status).

lDOM-leak = f\{**C-status**, $\underline{\boldsymbol{\mu}_{\mathbf{max}}}$\}

AA-leak = f\{**N-status**, **AAuptake**, **DINuptake**\}

Inorganic nutrient assimilations

Inorganic nutrients are sourced as phosphate (DIP), ammonium (NH4) and nitrate (NO3). The use of external nutrients depends on the substrate concentrations ([DIP], [NH4], [NO3]) and the respective uptake kinetics (uptake-V_{max}, affinity). The latter vary with the nutritional state of the organism, with uptake potential enhanced when nutrient-stressed and, at the extreme, shut down if nutrient-replete (i.e. uptake-V_{max} tends to zero at elevated nutrient status).

DIP assimilation = f\{**[DIP]**, $\underline{^{DIP}\mathbf{V}_{\mathbf{max}}}$, $\underline{^{DIP}\mathbf{V}_{\mathbf{max}}}$**/**$K_{DIP}$\}

$\underline{^{DIP}V_{max}}$ = f\{P-status, $\underline{\boldsymbol{\mu}_{\mathbf{max}}}$\}

$1/K_{DIP}$ = f\{**P-status**\}

The uptake of DIN is affected also by the P-status of the organism. The uptake kinetics for ammonium (NH4) provide for development of a much enhanced uptake capability over that for nitrate (NO3), with that development also enabled at a higher N-status. The latter results in ammonium being taken up ‘in preference’ to nitrate. There is no ‘inhibition’ term controlling NO3-assimilation by [NH4]; if the supply of ammonium from internal recycling plus external sources cannot meet the demand, then the ability to use nitrate is de-repressed.

DIN assimilation = f{**NH4-assimilation**, **NO3-assimilation**}

NH4-assimilation = f{**[NH4]**, $^{NH4}\mathbf{V_{max}}$, $^{NH4}\mathbf{V_{max}}$/K_{NH4}}

NO3-assimilation = f{**[NO3]**, $^{NO3}\mathbf{V_{max}}$, $^{NO3}\mathbf{V_{max}}$/K_{NO3}}

$^{NH4}V_{max}$ = f{N-status, P-status, μ_{max}}

1/K_{NH4} = f{**N-status**}

$^{NO3}V_{max}$ = f{N-status, P-status, μ_{max}}

1/K_{NO3} = f{**N-status**}

Exploitation of xdDOC

This material is complex C (i.e. no N nor P) which is degraded by extracellular digestion to liberate material taken up into the bacteria. The digestion of xdDOC is a function of the C-status of the bacteria.

xdDOC-assimilation = *f*{C-status, **[xdDOC]**,$^{xdDOC}\mathbf{V_{max}}$, K_{xdDOC}}

$^{xdDOC}V_{max}$ = *f*{**N:C-status**, μ_{max}}

Exploitation of xdDOM

This material is a complex of C,N,P. P associated with DOM is assumed to be cleaved to release DIP, due to the ubiquitous activity of phosphatases. The degradation of xdDOM_C and xdDOM_N is affected by the N:C of the material.

xdDOM_P digestion = *f*{P-status, **[xdDOM_P]**, $^{xdDOM\_P}\mathbf{V_{max}}$, K_{xdDOM_P}}

$^{xdDOM\_P}V_{max}$ = *f*{P-status, μ_{max}}

Chemoautotrophy (nitrification)

Nitrification, where enabled, develops as a function of poor C-status and requires elevated NH_4^+ ([NH4]) as the substrate. The process (which may be separated between different organisms in reality) is described as a single event here.

Nitrification = *f*{C-status, **[NH4]**, μ_{max}}

Supplementary Results

Table S1 Detail of the configuration of Exp.II. The table shows the intended inorganic nutrient concentrations, and also the actual initial concentrations in each flask (controls and treatments- Con and Treat; conditions - #1,#2,#3; triplicates within each condition – a,b,c). Note that nitrite was not an intended addition; it was present in the source seawater used as the base for the experiment and also as a contaminant in the added nitrate. See also **Table 1**.

Bottle	Target concentrations (µM)			Meaured initial concentration (µM)			
	NO_3^-	NH_4^+	PO_4^{3-}	NO_3^-	NO_2^- (*)	NH_4^+	PO_4^{3-}
Con#1 a	0.15	2.5	0.75	0.00	<0.01	2.13	0.75
Con#1 b	0.15	2.5	0.75	0.00	<0.01	2.22	0.79
Con#1 c	0.15	2.5	0.75	0.00	<0.01	1.92	0.68
Treat#1 a	0.15	2.5	0.75	0.02	0.04	2.41	0.81
Treat#1 b	0.15	2.5	0.75	0.03	0.04	2.71	0.87
Treat#1 c	0.15	2.5	0.75	0.03	0.03	2.66	0.84
Con#2 a	20	15	2.2	20.81	0.06	15.02	2.27
Con#2 b	20	15	2.2	21.29	0.14	14.83	2.28
Con#2 c	20	15	2.2	21.34	0.18	15.04	2.30
Treat#2 a	20	15	2.20	17.40	2.04	15.86	2.37
Treat#2 b	20	15	2.20	18.22	1.23	16.25	2.34
Treat#2 c	20	15	2.20	17.17	2.40	15.28	2.30
Con#3 a	20	75	5.9	20.62	0.07	73.78	5.79
Con#3 b	20	75	5.9	20.68	0.05	74.42	5.85
Con#3 c	20	75	5.9	20.57	0.10	73.11	5.81
Treat#3 a	20	75	5.90	16.66	2.86	75.92	5.84
Treat#3 b	20	75	5.90	16.95	3.49	75.50	5.97
Treat#3 c	20	75	5.90	21.41	2.73	76.37	6.05

Table S2 Results of ANOSIM tests used to explore the effect of DOM addition and ammonia concentration over time for Exp.II. Pairwise comparisons were used to explore the effect of ammonia concentration in more detail. See also **Table S1**, and **Fig.3**.

ANOSIM TEST	Rho	P	Pairwise	Rho	P
ALL DATA					
Test for differences between treatments (control vs DOM) (unordered)	0.511	0.001			
Test for differences between ammonium concentrations (2.5, 15 and 75 µM) (unordered)	0.274	0.001	2.5, 15	**0.361**	**0.001**
			2.5, 75	**0.291**	**0.001**
			15, 75	**0.18**	**0.002**
Test for differences over time (ordered)					
ALL time-points	0.769	0.001			
DAYS 0 – 12	0.726	0.001			
DAYS 166 - 348	0.264	0.002			
CONTROL: Test for differences between ammonium concentrations (unordered)					
ALL	0.25	0.001	2.5, 15	**0.245**	**0.003**
			2.5, 75	**0.192**	**0.009**
			15, 75	**0.278**	**0.01**
DAYS 0 – 12	0.136	0.051			
DAYS 166 – 348	0.373	0.001	2.5, 15	**0.354**	**0.002**
			2.5, 75	**0.354**	**0.003**
			15, 75	0.333	0.066
DOM: Test for differences between ammonium concentrations (unordered)					
ALL	0.293	0.001	2.5, 15	**0.448**	**0.001**
			2.5, 75	**0.371**	**0.001**
			15, 75	0.103	0.138
DAYS 0 – 12	0.341	0.001	2.5, 15	**0.443**	**0.002**
			2.5, 75	**0.391**	**0.002**
			15, 75	0.213	0.071
DAYS 166 – 348	0.23	0.034	2.5, 15	**0.245**	**0.013**
			2.5, 75	**0.192**	**0.046**
			15, 75	0.278	0.474

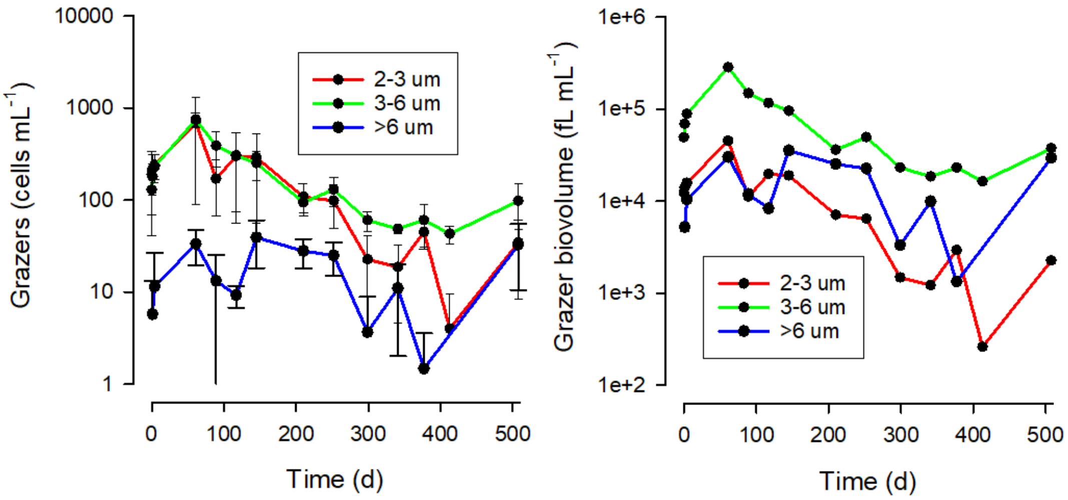

Fig.S1 Grazer abundance in Exp.I. These values are averages across 3 bottles for the '*community*' bottles. These are for microbes in suspension, excluding any that may have been attached to the bottle walls. These FlowCam data show changes in the protist abundance with respect to size structure; data are shown as cells and also as biovolume, the latter providing an index for biomass.

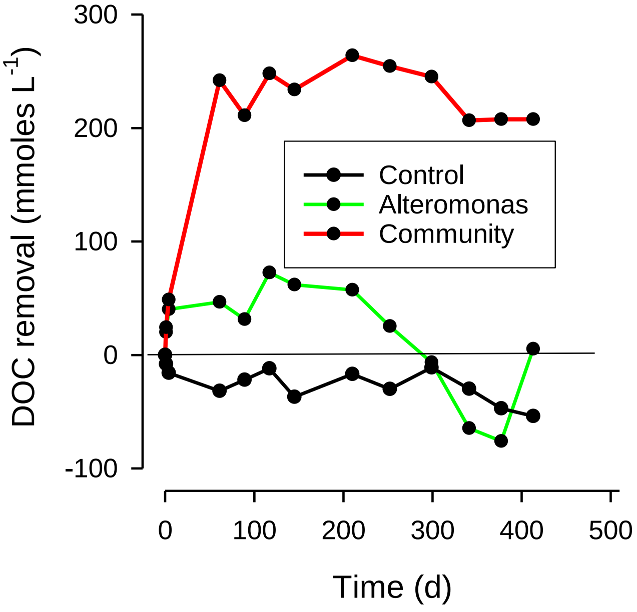

Fig.S2 Changes in net DOC removal in Exp.I. These data are shown for '*control*', '*Alteromonas*' and '*community*' bottles.

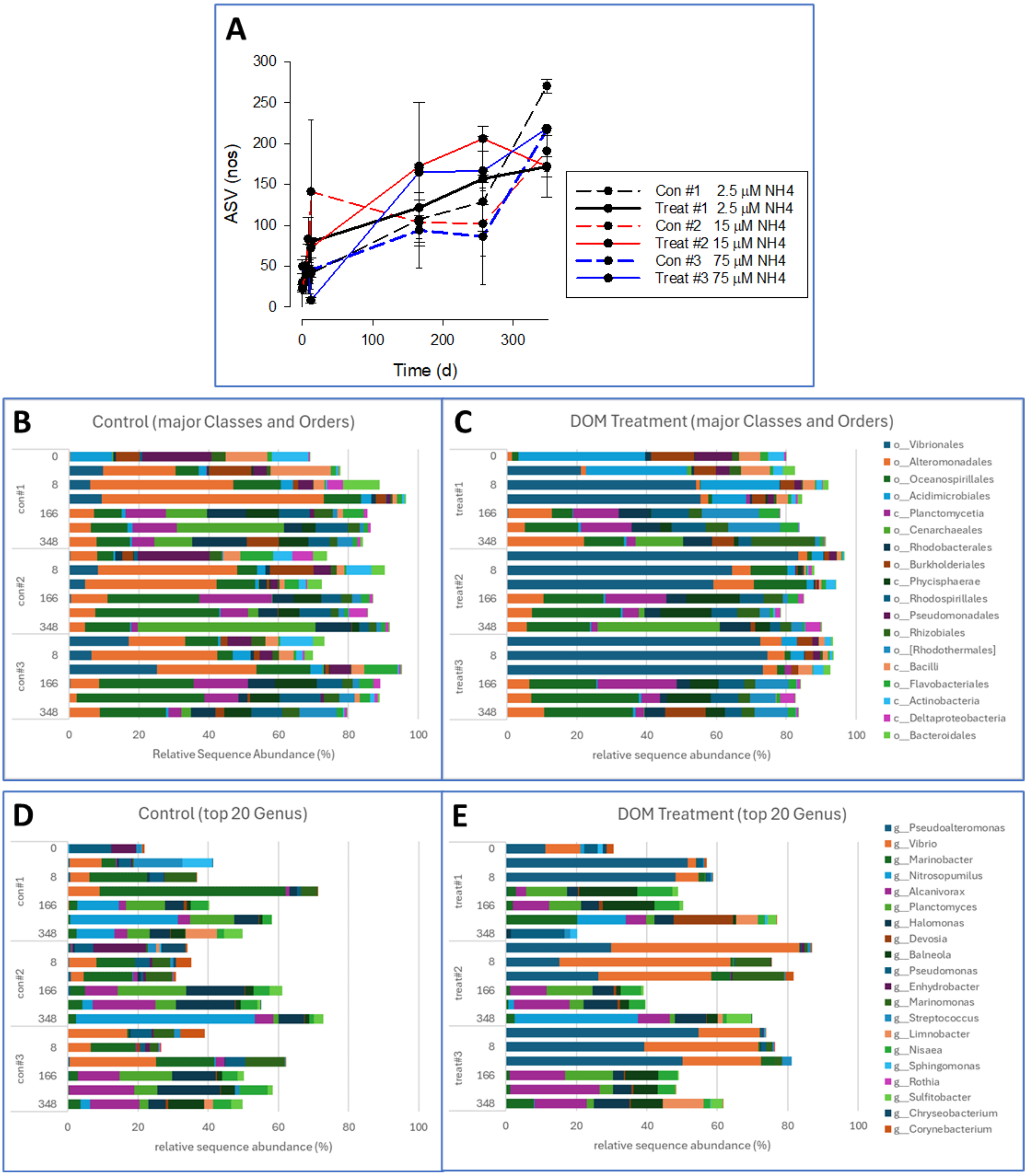

Fig.S3. **Changes to diversity and composition of the microbial community within control and DOM-amended treatments containing 2.5, 15 or 75 µM NH_4^+ in Exp.II.** A) shows increases to the numbers of 16S rRNA gene amplicon sequence variants (ASVs) in all treatments over time. 16S rRNA gene sequence data was grouped into Orders and Classes, and those with > 1% relative sequence abundance are plotted for A) control and B) DOM treatments. The 20 most abundant Genus present are compared for C) control and D) DOM treatments. See **Tables 1** and **S1** for conditions.

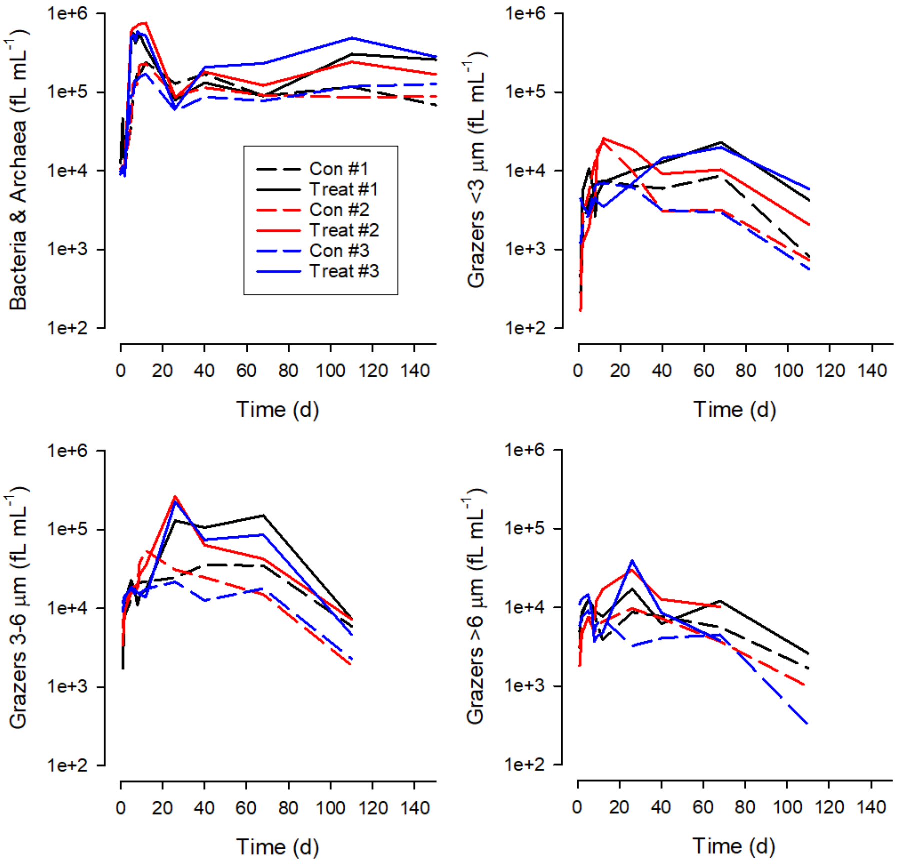

Fig.S4 Microbial community abundance in Exp.II. These values are averages across 3 bottles for each condition and are for microbes in suspension, excluding any attached to the bottle walls. The protist community data (from FlowCam) are displayed in 3 size fractions; bacteria and archaea were counted by analytical flow cytometry. Note the different Y-axis ranges. See **Tables 1** and **S1** for conditions.

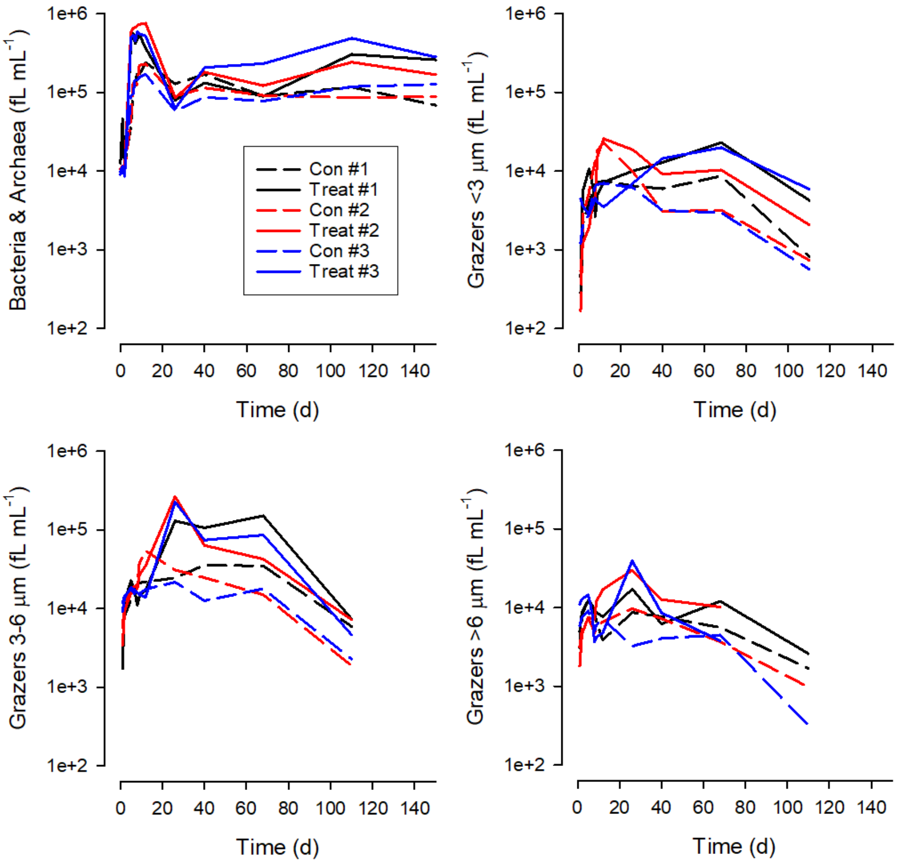

Fig.S5 The data shown in Fig. S4, transformed to biovolumes as an index for biomass. The assumed equivalent spheric diameters were: bacteria &archaea = 1µm; grazers <3 µm = 2µm; grazers 3-6µm = 4.5 µm; grazers >6 µm = 6 µm. The 3-6 µm grazer population attained a biomass of ca. 10% of that of the bacteria & archaea, with signs of decline in the bacteria/archaea prey with increased grazer presence between days 20-60 (see also **Fig.S4**). Assuming a C-biomass density of 200gC L^{-1}, a biovolume of 1e+5 fL mL^{-1} equates to ca. 1.43 µ moles C L^{-1}; this value is ca. 1% of the consumed DOC (**Figs. 2, S2**, and as simulated **Fig. 4**).

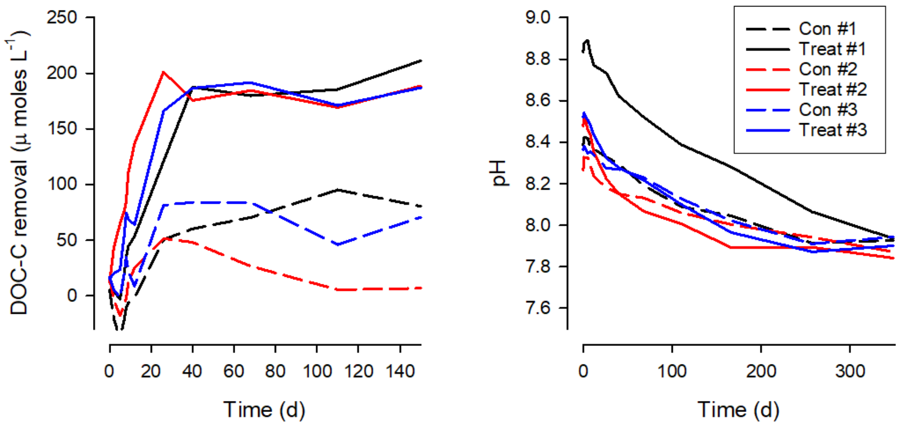

Fig.S6 Changes in DOC concentrations and pH in Exp.II. Note the different time axes. See **Tables 1** and **S1** for conditions.

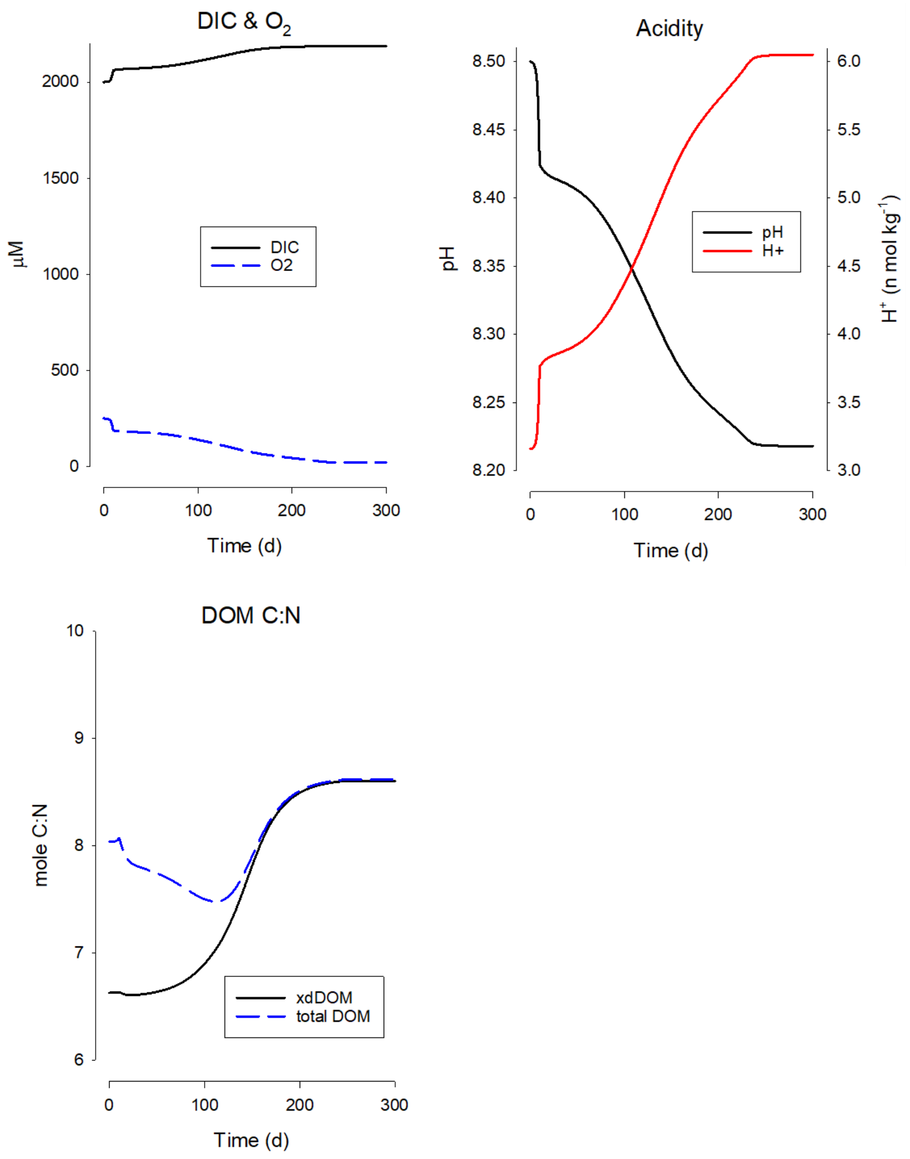

Fig.S7 Changes in DIC, O_2 , acidity and DOM C:N from the simulation shown in Fig.4.

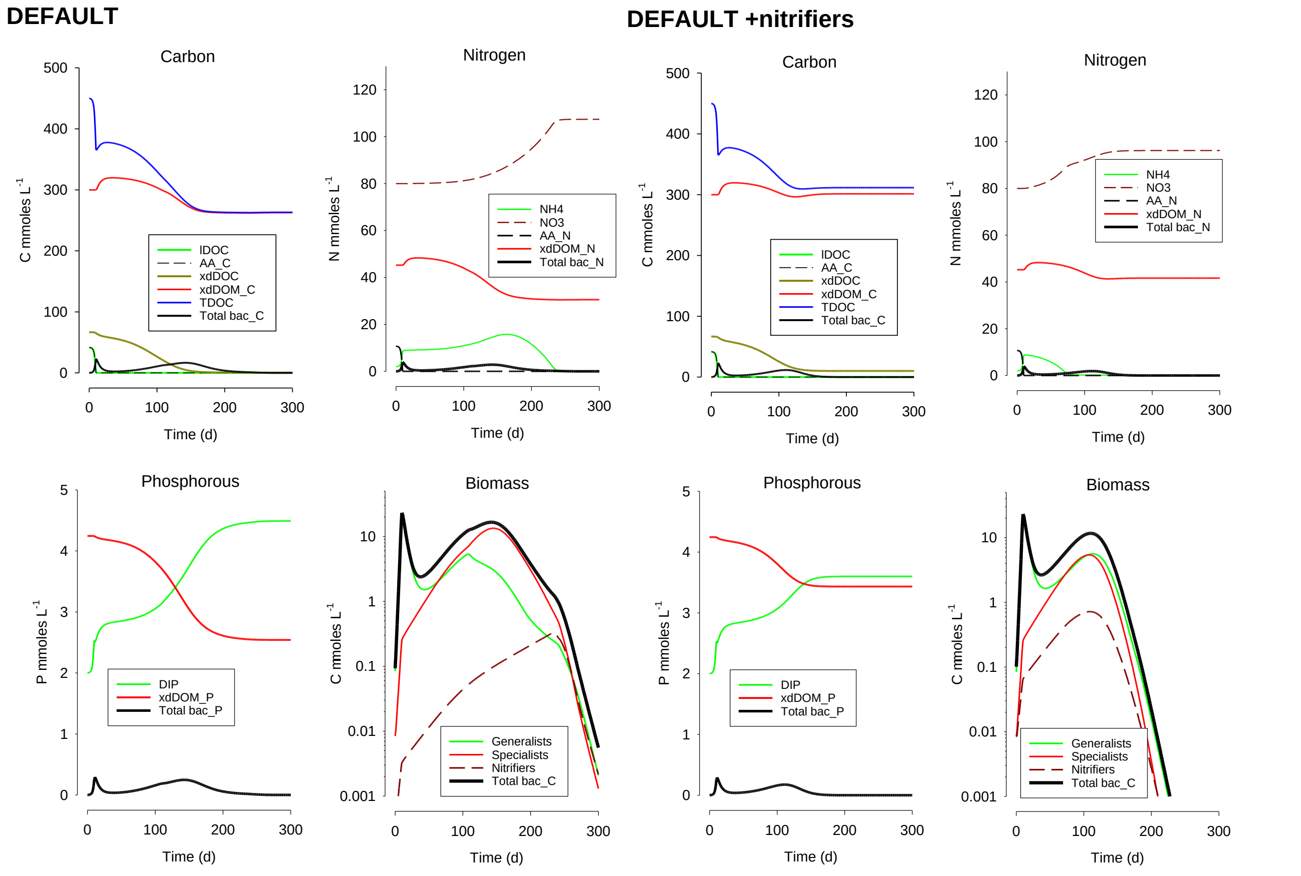

Fig.S8 Effect of commencing the simulation with higher abundance of nitrifiers. The lefthand panel ('Default') repeats the conditions of **Fig.4**, while the righthand panel ('Default +nitrifiers') starts with x10 the nitrifier biomass of the default. All other conditions remain the same. The addition of more nitrifiers withdraws NH_4^+ ('NH4') more rapidly, removing it as a N-source to support growth of heterotrophs exploiting DOC. Because NO_3^- is a much more expensive N-source to exploit than is NH_4^+, those heterotrophs cannot grow as well, affecting the ability to draw down the xdDOM_C.
